# Sigma-1 Receptor Antagonist (BD1047) Decreases Cathepsin B Secretion in HIV-Infected Macrophages Exposed to Cocaine

**DOI:** 10.1007/s11481-018-9807-4

**Published:** 2018-10-10

**Authors:** Omar Vélez López, Santhi Gorantla, Annabell C. Segarra, María C. Andino Norat, Manuel Álvarez, Richard L. Skolasky, Loyda M. Meléndez

**Affiliations:** 1 Department of Microbiology and Medical Zoology, School of Medicine, University of Puerto Rico Medical Sciences Campus, San Juan, 00936-5067 Puerto Rico; 2 University of Nebraska Medical Center, Omaha, NE 68198-5880 USA; 3 Department of Physiology, University of Puerto Rico Medical Sciences Campus, San Juan, 00921 Puerto Rico; 4 Department of Biology, University of Puerto Rico Bayamón Campus, Bayamón, 00959 Puerto Rico; 5 Department of Biology, University of Puerto Rico, Río Piedras Campus, San Juan, 00921 Puerto Rico; 6 Orthopaedic Surgery and Physical Medicine & Rehabilitation Director, Spine Outcomes Research Center, Johns Hopkins University, Baltimore, MD 21287 USA

**Keywords:** HIV-1, Cocaine, Sigma-1 receptor, Cathepsin B, HAND

## Abstract

Pathogenesis of HIV-associated neurocognitive disorders (HAND) is mediated through the infiltration of perivascular macrophages into the brain with the secretion of viral, neurotoxic and inflammatory proteins. One of these proteins is cathepsin B (CATB), a lysosomal cysteine protease that induces neuronal apoptosis, and increases in plasma and cerebrospinal fluid from HIV-1 infected patients (Cantres-Rosario et al. AIDS 27(3):347–356, 2013*).* Cocaine further potentiates CATB neurotoxicity in vitro and in vivo (Zenón et al. J NeuroImmune Pharmacol 9(5):703–715, 2014). Modulation of sigma-1 (Sig1R) by cocaine increases oxidative species, cytokines and other factors that promote lysosomal disruption. However, the role of Sig1R in CATB secretion and HIV-1 replication in macrophages exposed to cocaine is unknown. *We hypothesized that pharmacological modulation of Sig1R would alter CATB secretion from HIV-1 infected macrophages* in vitro *and* in vivo. To test our hypothesis, monocyte derived-macrophages (MDM) from HIV-1 seronegative donors were isolated, infected with HIV-1_ADA,_ and pretreated with Sig1R antagonist (BD1047) or Sig1R agonist (PRE-084) prior to cocaine exposure and followed for 3,6,9 and 11 days post-infection (dpi). Experiments in vivo were conducted using the HIV encephalitis mouse model (HIVE) with BD1047 treatments prior to cocaine for 14 days. Results demonstrate that in presence of cocaine, BD1047 decreases CATB secretion at 11 dpi, while PRE-084 did not have an effect. In the mouse model, BD1047 treatment prior to cocaine decreased CATB expression, cleaved caspase-3 an p24 antigen levels, reduced astrocytosis, but did not increase MAP-2 or synaptophysin. Results demonstrate that Sig1R plays a role in the modulation of CATB levels in HIV-1 infected MDM exposed to cocaine in vitro *and* in vivo.

**Electronic supplementary material:**

The online version of this article (10.1007/s11481-018-9807-4) contains supplementary material, which is available to authorized users.

**Graphical Abstract:**
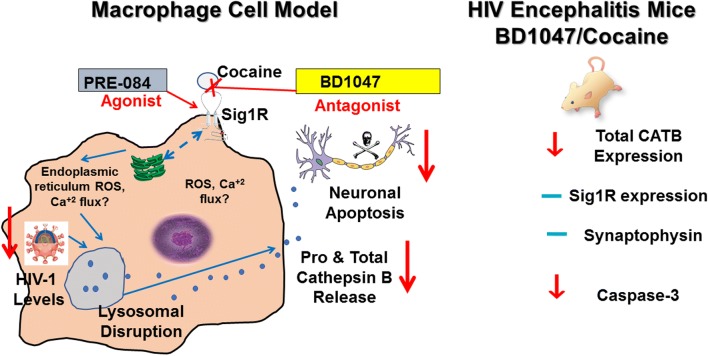
ᅟ

## Introduction

Human Immunodeficiency Virus (HIV-1) infection affects over 40 million people worldwide and more than 1.1 million people in the United States. Cocaine abuse is a major risk factor for the acquisition of HIV-infection and progression to AIDS (Larrat and Zierler 1993; Fiala et al. 1998). Despite combined antiretroviral therapy, HIV-seropositive cocaine users are more vulnerable to develop HIV associated neurocognitive disorders (HAND) than HIV-seropositive non-cocaine users. Considerable information suggests that peripheral extravasation of monocytes through the blood brain barrier (BBB) exacerbates HAND pathogenesis by secreting neurotoxic components, viral products, and inflammatory cytokines that induce damage to neural tissue. Cocaine, an addictive psychostimulant, not only increases HIV-1 viral replication but promotes inflammatory responses of diverse immune cells, including macrophages, that disrupt the blood-brain barrier (Dalvi et al. 2014), facilitates transmigration of infected monocytes into the brain (Yao et al. 2010), and amplifies the inflammatory genes response in macrophages (Atluri et al. 2016). The inflammatory products include cathepsin B (CATB), a cysteine protease found in lysosomes. In macrophages, CATB plays an important role in antigen processing and presentation, and when secreted, it can induce neuronal death (Rodríguez-Franco et al. 2012).

Previous results from our laboratory have demonstrated that extracellular secretion of CATB increases in HIV–1 infected macrophages when compared to uninfected cells (Rodríguez-Franco et al. 2012). An increase in CATB secretion was also observed in plasma of HIV seropositive patients, in *post-mortem* brain tissue of HIV patients with encephalitis, and with Alzheimer’s disease (Cantres-Rosario et al. 2013; Rodríguez-Franco et al. 2012). Furthermore, previous results demonstrate that cocaine potentiates CATB secretion in HIV-infected macrophages and increases neuronal apoptosis from 10 to 30% (Zenón et al. 2014). However, the mechanism by which cocaine further increases CATB secretion from HIV infected macrophages remained unknown.

A novel binding site of cocaine is the sigma 1 receptor (Sig1R). Originally classified as an opioid receptor due to its binding affinity for N-allylnormetazocine (SKF 10,047), it was subsequently characterized as a transmembrane chaperone protein. Sig1R is found abundantly in the endoplasmic reticulum where it modulates ion channels, regulates intracellular calcium concentrations and its related signaling molecules (Su et al. 2010). Sig1R binds a range of chemical compounds and has been the target of studies searching for novel therapeutic pharmacological strategies. Its role has been found to be mainly neuroprotective, dysfunction of the receptor has been implicated in the pathogenesis of several neurodegenerative diseases such as Alzheimer’s and Huntington’s diseases.

Cocaine binds to the Sig1R with an affinity of about 2-7 μM (Sharkey et al. 1988). It appears to act as an agonist, since Sig1R antagonists attenuate the physiological and cellular toxicities induced by cocaine (Yasui and Su 2016). Cocaine modulation of Sig1R has several physiological and cellular effects such as: increased HIV-1 replication in microglia (Gekker et al. 2006), augmented proinflammatory cytokines in microglia (Yao et al.*,*2010), and greater expression of transmigration molecules such as ALCAM and MCP-1(Yao et al. 2010, 2011) among other functions. However, other studies indicate that cellular effects after modulation of Sig1R by cocaine vary depending on the experimental settings 9 (Ka et al. 2016; Nixon et al. 2015; Weng et al. 2017. These findings demonstrate the versatility of Sig1R after cocaine modulation on diverse biological models.

Lysosomal proteases, such as CATB are responsible for the degradation of cellular components and are major players of autophagy under normal cellular functions (Vasiljeva et al. 2007). Rupture of lysosomes and extracellular secretion of lysosomal proteases such as CATB is dependent on pro-apoptotic pathways and on cellular events that are not involved in apoptosis such as calcium flux or reactive oxygen species (Repnik et al. 2012). In fact, antioxidants, such as dimethyl fumarate can prevent lysosomal disruption and decrease CATB secretion from HIV-infected macrophages (Rosario-Rodríguez et al. 2018).

Although no direct connection between Sig1R and CATB have been described, modulation of Sig1R by cocaine potentiates calcium dysregulation in cytosol, increases intracellular oxidative species from the endoplasmic reticulum, and augments mitochondrial dysfunction that may result in permeabilization of lysosomes (Hayashi and Su 2007; Boya and Kroemer 2008). Use of the specific Sig1R antagonist BD1047 abrogates the effect of cocaine on reactive oxygen species and on other toxic molecules (Yao et al. 2010).

Pharmacological approaches to modulate Sig1R effect on monocytes and macrophages and their possible contribution to HAND in vivo vary according to the animal model studied, and the agonists/antagonists used. For example, Roth et al. (2005) demonstrated that co-administration of the Sig1R antagonist BD1047 with cocaine abolished the increase in HIV-1 replication induced by cocaine in the huPBL- severe combined immunodeficiency (SCID) mouse model. In other studies, Yao et al. (2011) demonstrated that BD1047 treatment prior to cocaine administration reduced the expression of adhesion molecules such as ALCAM in mice. In similar studies, Yang et al. (2016) demonstrated that BD1047 attenuated cocaine-induced GFAP-astrogliosis in vitro. In similar experiments, treatment with the Sig1R antagonist BD1047 prior to cocaine abrogated reactive oxygen species in microglia (Yao et al. 2010), suggesting that it may prevent lysosomal permeabilization and cathepsin B exocytosis. While many functions of Sig1R pharmacology in HIV studies are still unknown, treatment with Sig1R antagonist BD1047 has been proposed as a plausible therapeutic measure in slowing the progression of HAND in HIV-infected cocaine users. Studies with the Sig1R agonist PRE-084 also demonstrates beneficial effects including the reduction of oxidative species, calcium flux and other inflammatory molecules within different cells (Szabo et al. 2014; Katnik et al. 2006).

In this study, we aimed to understand the role of Sig1R in CATB secretion in HIV-1 infected macrophages. *We hypothesized* that pharmacological modulation of Sig1R with antagonist or agonist would alter CATB secretion from HIV-1 infected macrophages in vitro and in vivo in the presence of cocaine. We also hypothesized that treatment with Sig1R antagonist BD1047 prior to cocaine will obliterate HIV-1 infection in macrophages, decrease CATB levels and its related neurodegenerative effects in vitro and in vivo. To examine if CATB secretion will be inhibited by Sig1R modulation, a specific antagonist (BD1047) prior to cocaine was tested in vitro and in vivo. In vitro studies with MDM from healthy donors infected with HIV-1 and treated with cocaine, BD1047 or both. The experimental design used for in vitro studies is based on previous publications from our group (Zenón et al. 2014; Rodríguez-Franco et al. 2012). The rationale for Sig1R agonist or antagonist experiments is based on the presumption that pretreatment of these drugs prior to cocaine exposure at (1,3,6,9 days post-infection) is more effective to block the action of cocaine on CATB secretion and HIV-1 infection. The selected concentration of Sig1R antagonist (BD1047) in absence of cocaine was based on titrations to ensure that it did not affect cell viability, did not promote HIV infection as determined by p24 antigen, and did not affect cathepsin B levels as shown in the *Supplementary Figures*. Future studies could address the role of this antagonist after exposure to cocaine.

In terms of virus and cocaine doses as well as the timeline used for antagonist treatment using the in vivo HIV encephalitis (HIVE) mice model, the design was based on Persidsky and Gendelman 1997. In this model, the pathology of HIV-1 including progressive inflammatory components of IL-1β, TNF-α and other proteins increases significantly untill days 12 through 14 post-injection. These cytokines could modulate CATB secretion and processing in different models (Guicciardi et al. 2001; Van de Veerdonk et al. 2011). The Sig1R antagonist dosage of BD1047 (20 mg/kg/weight) were based on Yao et al. 2011 and Roth et al. 2005 studies made on models of mice when administered prior to cocaine. The cocaine and antagonist (BD1047) concentrations used in HIVE were also based on previous pharmacodynamics/kinetics (PD/PK) studies (Lever et al. 2014). Our studies do not involve the measurement of withdrawal or tolerance of cocaine in these mice or any related physiological responses. But we are aware that other studies have considered these effects and have found that indeed 15 mg/kg/weight is able to induce cocaine tolerance and sensitization in rats (Bowen et al. 1993; Somkuwar et al. 2013).

## Materials and Methods

### Human Subjects

Eleven (*n* = 11) healthy female donors over 21 years of age were recruited for this study. A total of twelve (12) ACD blood tubes 8–10 mL were collected from each donor by a certified nurse. For the PRE-084/cocaine agonist studies, a total of three donors (*n* = 3) were used. For the PRE084 titration studies four (*n* = 4) donors were used. A total of four (n = 4) donors were used for BD1047 titration studies and BD1047/cocaine studies. The study was revised and approved by the University of Puerto Rico Institutional Regulatory Committee (IRB) (Protocol #0720416). Each donor willingly agreed to participate in the study and signed an informed consent.

### Isolation of Peripheral Blood Monocytes

Primary human macrophages were isolated from adherent peripheral blood mononuclear cells (PBMCs) obtained from the blood of healthy donors by Ficoll density gradient. PBMCs were cultured in T25 flasks (1 × 10^7^) or six well plates (5 × 10^6^) in complete monocyte media (fetal bovine serum 10% (FBS), human serum 1%, RPMI, Penicillin/Streptomycin 1%) (Sigma Aldrich, St. Louis, MO) and incubated at 37 °C in 5% CO_2_. Media was changed every 3 days, and after seven days, adherent cells were differentiated as ≥90% MDM as published previously (Rodríguez-Franco et al. 2012).

### Drugs

Cocaine hydrochloride and BD1047 dihydrobromide (a highly selective putative Sig1R antagonist) were purchased from Sigma Aldrich (St. Louis, MO.), PRE-084 (2-(4-Morpholinethyl)-1 phenylcyclohexanecarboxylate hydrochloride) at 99.9% purity was obtained from Tocris Pharmaceuticals (Avonmouth, Bristol, UK). The cocaine dose selected for this study (10 μM) was based on our previous studies and other viability assays (Zenón et al. 2014).

### HIV-1_ADA_ Infection of Monocyte Derived Macrophages

After 7 days in culture, MDM were inoculated with HIV-1 _ADA_ (University of Nebraska Medical Center) at a multiplicity of infection (MOI) of 0.1 in serum free media (Ross Park medium) (RPMI) (Ghorpade et al. 1998; Rodríguez-Franco et al. 2012). After overnight incubation (18–24 h), unbound viruses were removed and replaced with complete RPMI media.

### Treatments with Sig1R Agonist (PRE-084) and Antagonist (BD1047)

**Fig. 1:**
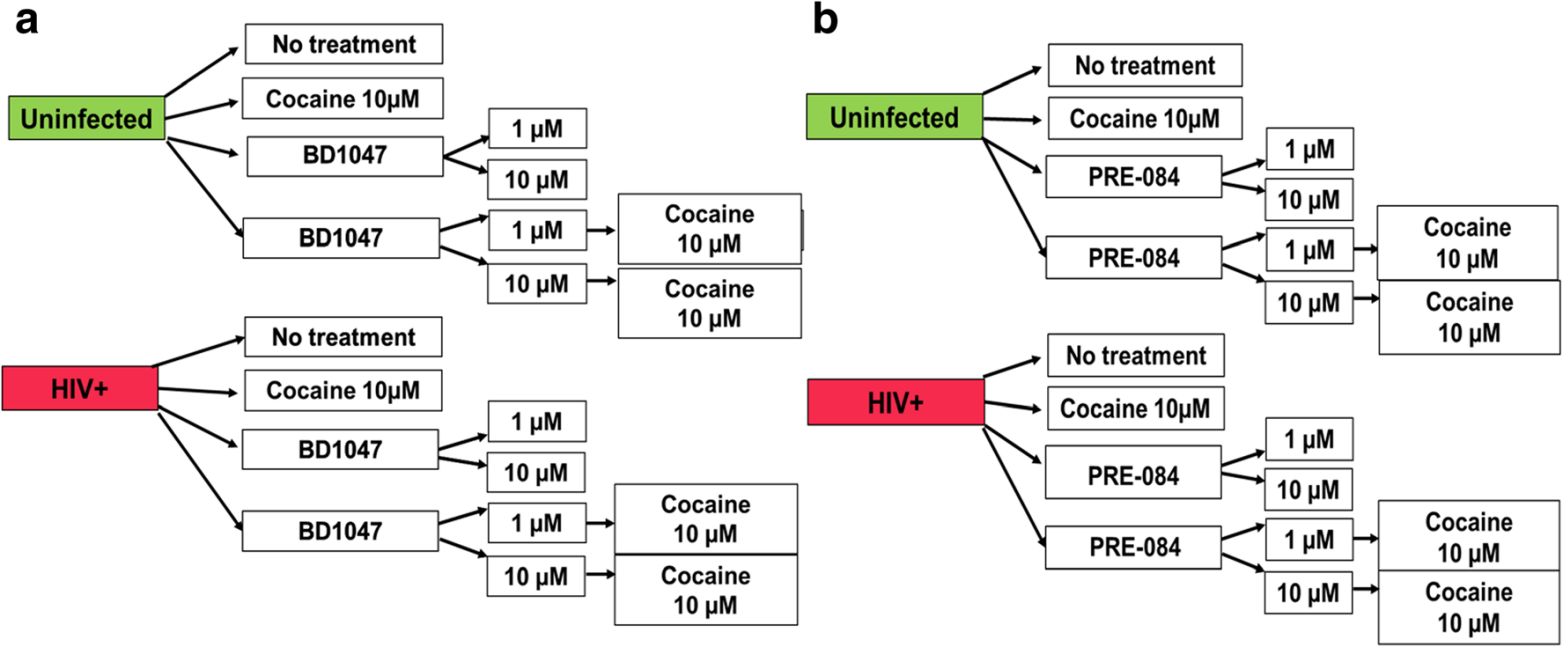
Diagram of Sig1R antagonist (BD1047) treatment to HIV infected MDM in presence of cocaine. (**a**) Uninfected and HIV-1 infected macrophages were treated with BD1047 1 or 10 μM for 1 h prior to each cocaine treatments for 3,6,9 days post-infection and compared to untreated controls. (**b**) Diagram of Sig1R agonist (PRE-084) treatment to HIV infected MDM in presence of cocaine. Uninfected and HIV-1 infected macrophages were treated with PRE084 1 μM for 1 h prior to each cocaine treatments for 3,6,9 days post-infection and compared to untreated controls

**Fig. 2:**
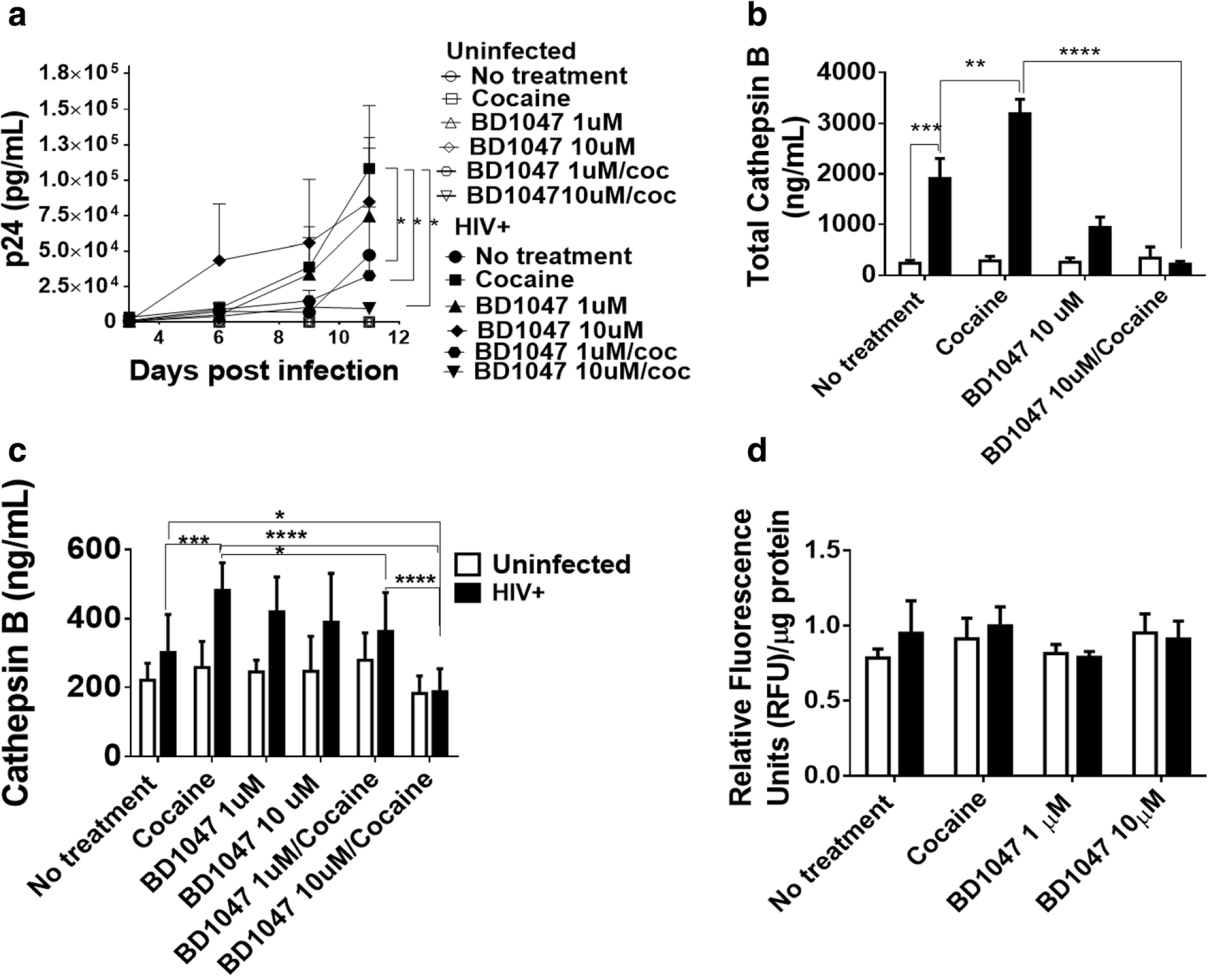
Effect of Sig1R Antagonist BD1047 in HIV replication, CATB secretion and activity by MDM in the presence of cocaine. Uninfected or HIV-infected MDM were treated with either antagonist (BD1047) or cocaine alone or in combination. (**a**) p24 (pg/mL) of MDM supernatants at 11 days post—infection, (**b**) Total CATB secretion (ng/mL) at 11 days post-infection (dpi), (**c**) CATB secretion (ng/mL) at 11 days post-infection (dpi), (**d**) CATB activity (RFU/μg of protein) at 11 days post-infection (*n* = 4). Two-Way ANOVA with Tukey’s post-hoc test (* *p* ≤ 0.05, *** *p* ≤ 0.001, **** *p* ≤ 0.001). Four donors depicted (n = 4)

At 1,3,6,9 days post HIV infection (dpi) MDM cultures were treated with cocaine (10 μM), with BD1047 (1 μM or 10 μM) or with both. BD1047 was added to the culture 1-h prior to cocaine or vehicle. This same procedure was followed for the PRE-084 studies. Treatment protocols with BD1047 and PRE-084 are detailed in Figs. 1 and 2 respectively. At days 3, 6 and 9 post-infection (dpi) half of the MDM culture supernatant was removed and media was replaced with fresh media containing cocaine and the inhibitor as described above. The supernatants were centrifuged at 1200 rpm for 10 min at 25 °C and aliquots stored at −80 °C (Rodríguez-Franco et al. 2012). At day 12 post-infection, all media was withdrawn, and serum free media (RPMI) was added. After 24 h, aliquots were removed and stored at −80 °C for neurotoxicity assays. The concentrations of BD1047 (1 & 10 μM) were selected based on two criteria: 1) a lack of increase in HIV-1 replication levels between infected MDM with BD1047 treatment (0.1–0.001 μM) at 11 days post-infection (Supplementary Fig. 1a); and 2) a lack of decrease in cell viability (MTT) obtained at 12 days post-infection in the presence or absence of cocaine (Supplementary Fig. 2a & 2b). In the same manner, PRE-084 concentrations were also selected based on its effect on HIV-1 levels (Supplementary Fig. 3a) and cell viability (Supplementary Fig. 4a & 4b) in the presence or absence of cocaine and in accordance with previous literature (Szabo et al. 2014).

### Determination of HIV Infection, CATB Secretion and Activity from Macrophage Supernatants

HIV p24 antigen and CATB concentration were measured from macrophage supernatants at 11 days post-infection by ELISA as described in previous studies (Rodríguez-Franco et al. 2012). To assess HIV-1 infection levels in macrophages after drug treatments, an HIV p24 antigen ELISA was performed according to the manufacturer’s instructions (Express BioTech, Maryland, USA). CATB secretion was determined at 3, 6, 9 and 11 days post-infection from cell culture supernatants using the Human Pro-Cathepsin B Quantikine ELISA kit (R&D Systems, Minnesota, USA). Total Human Cathepsin B Quantikine ELISA kit was also used (R&D Systems, Minnesota, USA). Macrophage supernatants from 11 days post-infection were analyzed for CATB in a Versa Fluor Fluorometer (Bio-Rad) (Bio Vision, San Francisco, CA) that contains 400 nm excitation and 505 nm emission filters**.**

### Cell Viability Assays and Total Protein Quantitation

Cell viability assays were performed in 96-well plates using Thiazolyl Blue Tetrazolium Blue reagent (MTT) (Sigma Aldrich, MO, USA) and measured at 450 nm in a Dynex MRX Revelation Microplate Reader (Chantilly, VA). Readings were plotted in Excel Microsoft software; experimental groups were standardized to non-infected and non-treated controls using the following equation:$\frac{\boldsymbol{experimental}}{\boldsymbol{control}\ }\ \boldsymbol{x}\ \mathbf{100}$. Results were reported as the percent viability (%) from control. Bicinchoninic acid assays for total protein quantitation from MDM supernatants were performed according to manufacturer’s instructions (Bio Rad, La Jolla, CA). Samples from different donors were assayed in triplicates and read at 450 nm in a Dynex MRX Revelation Microplate Reader (Chantilly, VA).

### Expression of Sig1R, CATB, and Neuronal Damage Markers In Vitro and In Vivo

To determine the effect of cocaine on Sig1R and CATB intracellular levels, macrophages were lysed at 12-day post-infection (12 dpi) using a lysis buffer containing 5 mM Tris-HCl at pH 8, 0.1 Triton X-100 and proteinase inhibitors (Sigma Aldrich, St. Louis, MO). For quantification of Sig1R, CATB expression, and neurogenesis marker (MAP-2) in the HIV-encephalitis (HIVE) mice, approximately 2 mm^3^ of dissected *striatum* sections were weighed (40–85 mg), homogenized and the cytosolic fraction used for protein determinations using the Mem-PER Total Protein Extraction Kit (Thermo Fischer Scientific, USA). Thirty (30 μg) micrograms of protein from lysates and homogenates were placed on BioRad Mini-Protean SDS-polyacrylamide gels (15–20%), transferred onto difluoride membranes (PVDF) and incubated with a monoclonal primary antibody to Sig1R (Santa Cruz Biotechnology, 1:200); CATB (Sigma Aldrich, 1:100), MAP-2 (Cell Signaling, 1:500). Images were acquired and analyzed using Image Lab™ software (Bio-Rad). Band intensity was quantified by densitometry and normalized using the GAPDH band (Santa Cruz Biotechnology, 1:100) for each lane (Image Lab Software, Bio Rad Laboratories, Hercules, CA). Membrane chemiluminescence was analyzed using the Bio Rad Chemi-Doc instrument at different exposure times ranging from 15 s to 80 s (Image Lab Software, Bio Rad Laboratories, Hercules, CA). Membranes were stripped incubating in Restore Plus Mild Western Blot Stripping Buffer (Thermo Fisher Scientific, Waltham, MA) for 30 min at 37 °C followed by washing, blocking, and re-probing with different antibodies.

### Human Neuroblastoma Cell Cultures for Apoptosis Assays

SK-N-SH (HTB-11) cells (human neuroblastoma cell line, ATCC), were grown and plated in 4-well chamber slides (Fisher Scientific, Suwannee, GA) at a density of 2 × 10^5^ cells per well and cultured in Eagle’s MEM (EMEM) supplemented with 10% fetal bovine serum (FBS), 1% sodium pyruvate and 1% non-essential amino acids (all from Sigma) and incubated for 3–5 days at 37 °C, 5% CO_2_ until 75–80% confluence. HTB-11 neurons were used for future apoptosis (TUNEL) assays after exposing them to macrophage conditioned media (MCM) with each of the treatments.

### In Situ Cell Neuronal Death (TUNEL) Assay

This procedure was performed following previously described protocols (Zenón et al. 2014). Briefly, confluent SK-N-SH neurons were washed with Phosphate Buffered Saline (PBS) and incubated with MDM conditioned medium (MCM). The MCM consisted of fresh serum-free supernatants from HIV-infected, uninfected, uninfected cocaine-treated, HIV-infected cocaine-treated, uninfected BD1047 prior to cocaine and infected prior to cocaine MDM from four different donors and diluted 1:4 with plain EMEM. The MCM was added with or without the specific cathepsin B inhibitor (CA-074) at 50 μM. In addition to CA-074, a mouse anti-human monoclonal cathepsin B antibody (Sigma) was used at 1:500, which binds 50X of secreted cathepsin B and inhibits the enzyme. For apoptosis determination with TUNEL assay, SK-N-SK were grown in 8-well chamber slides at a density of 2 × 10^5^ cells per well and incubated at 37 °C, 5% CO_2_ until 75–80% confluence. MCM was added to confluent neurons and incubated at 37 °C, 5% CO_2_ for 24 h. The next day, neurons were washed with PBS and fixed with 4% paraformaldehyde (PFA) for 1 h. To quench auto-fluorescence, fixed neurons were incubated with 3% hydrogen peroxide with methanol for 10 min and permeabilized on ice with 0.1% TritonX-100 in 1.0% sodium citrate for 10 min. In situ cell death detection Kit; also named as TUNEL (TdT-mediated dUTP-X nick end labeling) (ROCHE®) was performed by incubating neurons with TUNEL reaction mix for 1 h at 37 °C in a humidity chamber in the dark. Cells were washed 3 times with PBS and covered with a coverslip using anti-fade mounting media (Vectashield®). The positive control consisted of cells incubated with recombinant DNase I (30 U/Ml) for 10 min at room temperature to induce DNA breaks. The negative control consisted of cells incubated with the label solution without the enzyme. An Olympus microscope Z-100 series with LSM 510 under an excitation wavelength of 488 nm, 20× magnification was used to view the samples. Analysis of percentage of apoptosis was determined using the Image J program software (National Institutes of Health).

### Mice Rearing and Handling

Four (4) week old female CB-17/Icr Crl-SCIDbr mice from Charles River Laboratory (Wilmington, MA) were maintained in sterile micro isolator cages in a temperature and humidity-controlled room under pathogen-free conditions with food and water provided ad libitum in the animal facilities of the University of Nebraska Medical Center (UNMC). All procedures with animals were previously approved by the IACUC committee of the University of Nebraska Medical Center (UNMC) and are in accordance with the ethical guidelines for the use and care of laboratory animals set forth by the National Institutes of Health (NIH) and Office of Laboratory Animal Welfare (OLAW).

### HIV Encephalitis (HIVE) Mouse Model

Human monocytes from three donors (*n* = 3) obtained by centrifugal elutriation were maintained in suspension cultures. After 7 days, post-differentiation, human monocyte-derived macrophages (MDM) were infected with HIV-1_ADA_ 0.1 MOI overnight. These MDMs were used in our in vivo experiments as described below. CB-17 mice were anesthetized using a ketamine/xylazine solution (100 mg/kg ketamine, 16 mg/kg of xylazine, i.p.). They were then inoculated with 5 × 10^5^ cells (in 5 μL) of infected or uninfected MDM’s as described by Persidsky and Gendelman 1997. MDMs were infused (Hamilton syringe, 26 gauge) intracranially into the *striatum* (anterior basal ganglia) of CB-17 mice using a stereotactic apparatus (Stoelting, Wood Dale, IL). The coordinates used were AP: 3.5 mm; ML 3.5 mm, DV 4.0 mm relative to Bregma. Injected mice were left undisturbed for a day. The next day, and for the following 13 days, mice were injected with normal saline (PBS) (non-cocaine controls) i.p. or with cocaine hydrochloride (15 mg/kg). In addition, a separate group of animals received BD1047 (20 mg/kg) 1 h prior to cocaine injections. Animals were divided into six (6) groups with three (*n* = 3) mice in each group: uninfected saline, uninfected cocaine, uninfected BD1047 + cocaine, infected saline and infected cocaine and infected BD1047 + cocaine. On day fourteen (14), four (4) hours after the last cocaine injection, mice were sacrificed, the brains removed, and the *striatum* manually dissected with a scalpel. Tissues were frozen in ethanol at −80 °C for protein assays. Brain sections from three (n = 3) uninjected mice served as controls. Other tissues were fixed in paraformaldehyde for future immunohistochemistry procedures.

### Immunohistochemistry of HIVE Mice Tissues

The HIV Encephalitis mice serial coronal sections were processed at UNMC, embedded in paraffin, and cut coronally (10 μM) thickness until the needle trace was found in each mouse brain tissue. For this procedure, three brain tissues of each mouse were processed for the following treatments: HIV-1 infected tissues, HIV+ cocaine infected tissues and HIV + BD1047 prior to cocaine tissues. The contralateral region to the injection site of the human MDM served as the uninfected control due to the static nature of the model and the inability of HIV-1 infection to migrate to the contralateral site in mice (Persidsky and Gendelman 1997).

The procedure for fluorescent staining of each of the samples were done at UPR-MSC in accordance with a previous study (Zenón et al. 2014). The primary antibodies used included anti human antibodies against cathepsin B (CATB) (1: 100; Sigma Aldrich Company), Ionized calcium binding adaptor protein (Iba-1) (1:100, Wako, USA), Synaptophysin (1:100, Abcam MO, USA), Cleaved Caspase-3 (Abcam MO, USA), p24 HIV-1 capsid antigen (1:50, Agilent Company, CA USA) & glial acidic fibrillary protein (GFAP) (1:100, Abcam MO, USA). These antibodies are known to crossreact with mice antigens. The secondary antibodies used were fluorescein isothiocyanate 488 (FITC) (1:200; Invitrogen Thermo Scientific CA, USA) and Rhodamine Red 545(1:200; Invitrogen Thermo Scientific CA, USA). Sections were visualized under a Nikon Eclipse E400, with a Nikon camera DS-Qi2 and Fluorescence X-Cite Series 120.

Brain sections (three fields of view) were chosen randomly and analyzed blindly by the investigator for each labeled protein using 20X & 40X objectives. The NIS Elements BR Program version 6.1 (Nikon, USA) was used to quantify single count measurements. Each of the respective staining intensities was counted with the software individually. A ratio between stained cells (stained with the respective antibody) vs total cells (stained with DAPI) was calculated for each antibody and treatment $\frac{\boldsymbol{Sspecific}\ \boldsymbol{antibody}\ \boldsymbol{staining}\ }{\boldsymbol{DAPI}\ \boldsymbol{staining}\ }$). Colocalization studies were done by superimposing images of each fluorophore (green and red color) and divided by total cell count **(**$\frac{\boldsymbol{Singlecount}\ \boldsymbol{p}\mathbf{24}\ \boldsymbol{p}\boldsymbol{er}\ \boldsymbol{Iba}+\boldsymbol{cell}}{\boldsymbol{Singlecount}\boldsymbol{ofDAPI}}\boldsymbol{x}\mathbf{100}$**)** and reported as percentage (%) of p24 colocalized with Iba+/DAPI. Photo sections were viewed at a minimum resolution of 1200 pixels/in. Integrated density measurements were done using the Image J program software (National Institutes of Health) and quantified accordingly.

### Immunohistochemistry of *Post-Mortem* Human Brains

Paraffin embedded samples of *post-mortem* human brain tissue of the *basal ganglia* were obtained from the National Neuro AIDS Tissue Consortium (NNTC). Serial sections of formalin-fixed, paraffin-embedded basal ganglia (10 μM thickness) from patients or normal controls were placed on electromagnetically charged glass slides. The primary antibodies used included CATB (1: 100; Sigma Aldrich Co.), and Sig1R (1:200; Novus Biologicals). Secondary antibodies used were fluorescein isothiocyanate 488 (FITC) (1:200; Vector Laboratories) or Rhodamine Red 545(1:200; Vector Laboratories).

Sections were visualized under a Nikon Eclipse E400, with a camera SPOT Insight QE and Fluorescence X-Cite Series 120. Brain sections (three fields of view) were chosen randomly and analyzed blindly by the investigator for each labeled protein using a 40X objective. The Spot Software was used for merged analyses and the IMARIS imaging Software was used for single counting using total intensity count. In total intensity count, mean intensities were measured for DAPI staining as well for red and green staining. Each of the respective staining intensities was counted with the software individually. A ratio between intensity of color and DAPI staining was made per each treatment ($\frac{\boldsymbol{Mean}\ \boldsymbol{intensity}\ \boldsymbol{of}\ \boldsymbol{color}\ \boldsymbol{spot}\ \boldsymbol{count}\ }{\boldsymbol{Mean}\ \boldsymbol{intensity}\ \boldsymbol{of}\ \boldsymbol{DAPI}\ \boldsymbol{spot}\ \boldsymbol{count}\ }$). Photo sections were viewed at a minimum resolution of 1200 pixels/in. and contrast adjusted slightly and evenly for all sections using the Adobe Photoshop software version 7 (San José, CA, USA).

### Statistical Analyses

Data were analyzed using the Graph Pad Prism Software Inc. versions 6.1 & 7.1 (La Jolla, CA) and Excel Microsoft 2007 program (Microsoft, USA). After testing normality by Shapiro-Wilk Test, several statistics were used for each of the assays. Paired data sets were grouped and analyzed with a multiple comparison Two Way-ANOVA test using Tukey’s as a *post-hoc* analyses with a 95% confidence as statistically significant (*p ≤ 0.05, **p ≤ 0.01, ***p ≤ 0.001, ****p ≤ 0.0001). Also, to account for the auto-correlation of CATB and p24 expression within each donor across experimental conditions, we employed a repeated measure analysis of variance (MANOVA) to determine the influence of HIV infection, cocaine, and BD1047 on expression of CATB and p24. We included main effects of HIV infection (negative and positive), cocaine (absent and present), and BD1047 (1 μM and 10 μM concentration). We included two- and three-way interaction effects to determine how the influence of one factor on expression may be mediated by another factor. Graphical representations of these two- and three-way based predictive models of interactions were examined to describe the significant interaction terms between factors. Repeated measures ANOVA was computed using Stata, version 14.2 (Stata Corporation, College Station, TX). Level of statistical significance was set at *p* ≤ 0.05 (*p ≤ 0.05, **p ≤ 0.01). Statistical raw data is available if requested.

## Results

### Sig1R Selective Antagonist BD1047 Prior to Cocaine Treatments Reduces HIV-1 Infectivity and CATB Secretion in Macrophages

The Sig1R antagonist BD1047 dihydrobromide was titrated from 0.001 μM to 10 μM to determine the best concentration that does not affect viability, HIV-1 infectivity, or CATB secretion in macrophages prior to testing antagonist/cocaine treatments. After 11 days post-infection, results demonstrate that 10 μM and 1 μM had no effect on HIV-1 infection while lower concentrations increased HIV-p24 levels at the same time point (Supplementary Fig. 1a) or CATB levels (Supplementary Fig. 1b). Therefore, we selected these two concentrations for antagonist/cocaine assays since these do not affect HIV-1 infection or cell viability in absence or presence of cocaine (Supplementary Fig. 1 & 2).

For antagonist/cocaine assays, cells were pretreated with BD1047 (1 or 10 μM) for one (1) hour prior to cocaine exposure and supernatants evaluated for HIV infectivity (p24 levels) at 3,6,9 and 11 days post-infection. CATB secretion was evaluated from MDM supernatants by ELISA at 11 days post-infection in accordance with previous studies. Treatment scheme is described in (Fig.1a). The same procedure was followed with Sig1R agonist PRE-084 and subsequent cocaine treatments (Fig.1b).

Experiments performed using an average of four different donors (*n* = 4) demonstrate that treatment of infected MDM with BD1047 10 μM in the presence of cocaine significantly decreased HIV-1 p24 levels (Fig.2a) and CATB secretion (Fig.2b and c) when compared to cocaine treated MDM. Using repeated measures ANOVA, there were significant interactions between HIV and cocaine (***p ≤ 0.001) and between HIV, cocaine and BD1047. No significant effects on CATB activity in any of the treatments were found (Fig.2d) (*p* ≥ 0.05). These results demonstrate that BD1047 blocks the action of cocaine on CATB secretion. In terms of infection, BD1047 blocks the effect of cocaine on HIV infection as demonstrated by significant decreases in p24 antigen levels (*p* = 0.0026). For non-treated and infected MDM, BD1047 increase in p24 levels in small amounts, although it is not significant (Fig.2a). This was not observed on CATB levels (Fig.2b and c). Predictive parameters of HIV infection, BD1047 and cocaine presence and absence as well as CATB using Stata Statistics Models are seen in (Supplementary Fig. 3a & 3b).

### Sigma-1 Receptor Selective Agonist PRE-084 Decreases HIV Infectivity but Had no Effect on CATB Secretion in HIV-1 Infected Macrophages Exposed to Cocaine

**Fig. 3:**
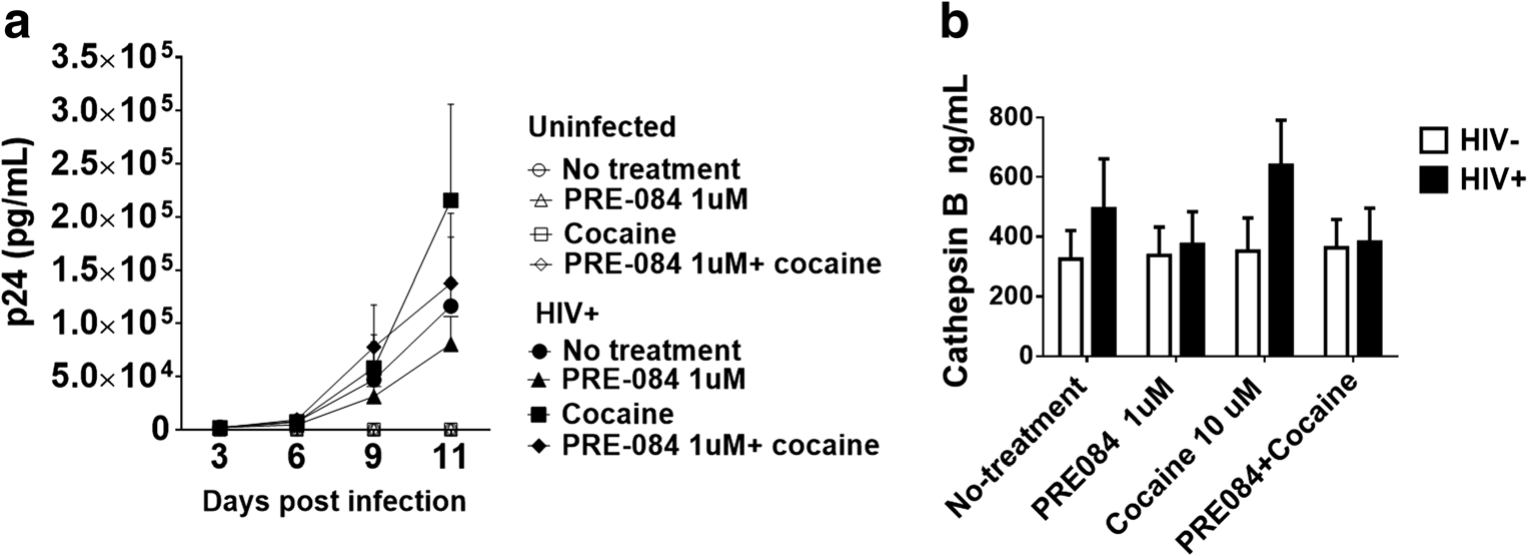
Effect of Sig1R agonist in HIV-1 replication and CATB secretion by MDM in the presence of cocaine. Uninfected or HIV-infected MDM were treated with the Sig1R agonist (PRE-084) in presence or absence of cocaine. (**a**) HIV-1 p24 (pg/mL) of infected MDM supernatants at 11 days post—infection, (**b**) CATB secretion (ng/mL) at 11 days post-infection. Two-Way ANOVA with Tukey’s post-hoc test. Three donors depicted (*n* = 3)

Previous studies indicate that PRE-084 (Sig1R agonist) may have neuroprotective antioxidant properties and a beneficial role in HIV-1 infection (Nguyen et al. 2014; Katnik et al. 2006: Allahtavakoli and Jarrott 2011), experiments were also performed to test its effect on HIV-1 infection levels and CATB secretion. Initial studies were performed to titrate the agonist in absence of cocaine from 0.0001–1 μM. Results show that HIV-1 infection (p24 levels) increase with lower concentrations of Sig1R agonist in a dose dependent fashion (0.0001, 0.001, 0.1 μM) when compared with infected MDM controls (Supplementary Fig. 4a). The concentration of 1 μM did not affect HIV replication but decreased CATB secretion in MDM in the absence of cocaine (Supplementary Fig. 4b) without affecting cell viability in presence or absence of cocaine (Supplementary Fig. 5). For these reasons, 1 μM of PRE-084 was selected for further testing in presence of HIV-1 and cocaine. When PRE-084 1 μM was added in presence of cocaine, it did not change HIV-1 infection levels in MDM when compared to HIV-infected MDM treated with cocaine (Fig. 3a), neither had any effects on CATB levels (Fig. 3b).

### Neuronal Apoptosis (TUNEL) Assay of HTB-11 Neurons Treated with MDM Conditioned Media (MCM)

**Fig. 4:**
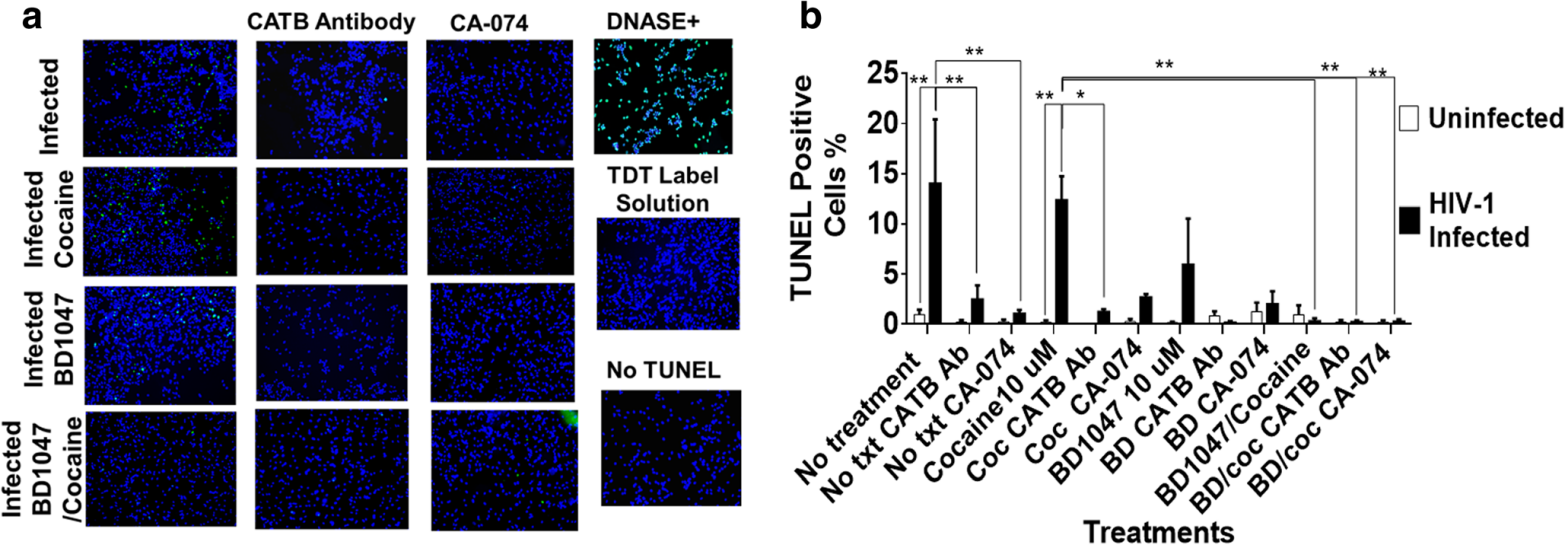
**a** In Situ Cell Death (TUNEL) Assay of HTB-11 Neuroblastoma Cell Lines treated with MDM Infected (HIV+) Conditioned Media. (**a**) Green Fluorescence means cell death of HTB-11 neurons on regards of DNA damage. Nucleus is stained with DAPI (blue color). Controls involve neurons treated with DNase and its related negative controls. Results representative from four donors (*n* = 4). (**b**) Quantification of In Situ Cell Death (TUNEL) Assay of HTB-11 Neuroblastoma Cell Lines treated with MDM Uninfected and Infected Conditioned Media. Results are presented as TUNEL Positive Cell percentage (%) per DAPI. Representative from four donors (*n* = 4). Statistical Analyses were done with Two-Way ANOVA using the Tukey’s Post-Hoc test and Multiple Comparison Test (*p ≤ 0.05, **p ≤ 0.01). A monoclonal antibody against CATB and a specific chemical inhibitor (CA-074) were used for determining CATB specificity

The HTB-11 neurons treated with HIV-infected MCM showed greater percentage of apoptosis compared to neurons exposed to uninfected MCM (**p ≤ 0.01) (Fig. 4) & (Supplementary Figure 5). Cocaine further increased this effect on neurons treated with infected MCM (*p ≤ 0.05). Pretreatment of neurons with BD1047 treated MCM prior to cocaine administration significantly reduced apoptosis when compared with the non-BD1047 cocaine group (**p ≤ 0.01). Treating MCM with either a monoclonal antibody or the specific CATB inhibitor, CA-074 reduced apoptosis when compared with their control counterparts. Positive controls such as DNASE+ confirmed that the assay worked properly. These results demonstrate that Sig1R antagonist (BD1047) prior to cocaine significantly abrogates cocaine’s action on neuronal apoptosis induced by HIV- infection.

### Intracellular Expression of Sig1R and CATB in HIV-1 Infected Macrophages Exposed to Cocaine

Sig1R was measured from MDM lysates to determine if there was an increase on intracellular expression of this receptor on HIV-1 infected macrophages exposed to cocaine at 12 days post-infection. Blots of intracellular proteins demonstrate no significant differences in the expression of Sig1R as well as of CATB from three donors (*n* = 3) compared to uninfected or untreated MDM controls (Supplementary Fig. 7). These results confirm previous observations that intracellular levels of CATB do not change on HIV-infected macrophages after cocaine exposure (Rodríguez-Franco et al. 2012; Zenón et al. 2014).

### BD1047 Prior to Cocaine Reduces CATB in HIV Encephalitis Mouse Model (HIVE) Anterior Basal Ganglia *striatum* Tissues but has no Effect on Sig1R or MAP-2 Expression

**Fig. 5:**
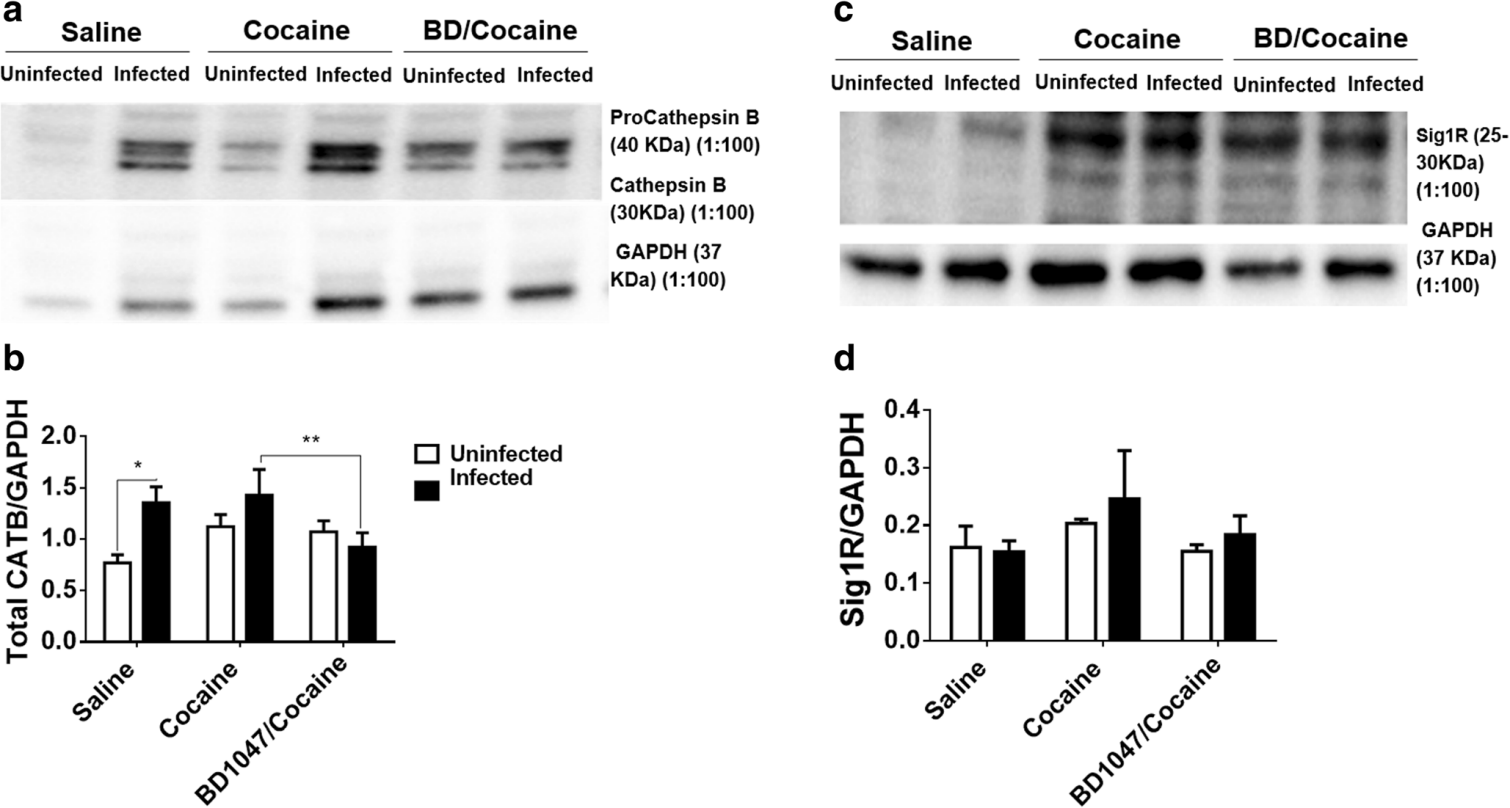
CATB and Sig1R expression in the *striatum* of HIVE mice model. **a** Western Blot of brain sections from HIVE mice containing the injected area to analyze CATB expression after 14 days of HIV-1 infected MDM injection, cocaine treatments or BD1047/cocaine treatments (*p ≤ 0.05) (n = 4 per group). **b** Normalization of CATB from mice using GAPDH as an internal control**.** Four mice (n = 4) depicted. **c** Sig1R expression in the *striatum* of HIVE mice. Western Blot of brain sections from HIVE mice containing the injected area to analyze Sig1R expression after 14 days of HIV-1 infected MDM injection, cocaine treatments or BD1047/cocaine treatments (*p ≤ 0.05) (n = 4 per group) (**d**). Normalization of sigma-1 receptor from mice was done using GAPDH as an internal control. Four mice (n = 4) depicted. (* *p* ≤ 0.05, **p ≤ 0.01)

To determine if CATB and Sig1R expression are affected by HIV infection and cocaine exposure in vivo*,* experiments were conducted using the HIVE mice model that resembles the typical neuropathology of HIV-1 (Persidsky and Gendelman 1997). Results demonstrate that injection of HIV-1 infected human macrophages increases CATB expression in the *striatum* of mice by Western Blot (p* ≤ 0.05). Cocaine treatment of HIV-infected mice did not increase CATB over uninfected mice. Cocaine treatment of HIV-infected mice did not increase CATB over HIV-infected untreated group (Fig. 5a and b). However, treatment of mice with BD1047 prior to cocaine reduced CATB expression when compared with the cocaine treated group (**p ≤ 0.01). Cocaine treatment or HIV-infection did not influence the expression of Sig1R in any of the mice groups (Fig. 5c and d). Unfortunately, we did not have enough mice tissues to include BD1047 control and relate its antagonist effect on CATB and Sig1R expression. To determine the potential effect of Sig1R ligands on the neuropathology of HIVE mice, cytoskeletal microtubule associated proteins-2 (MAP-2) isoforms of high and low molecular weights, proteins involved in dependent dendritic and synaptic plasticity in the adult brain (Kaech et al. 2001), were analyzed using Western blots. We found that HIV-infection decreased low molecular weight MAP-2 isoforms C and D expression levels. Cocaine seemed to decrease these MAP-2 isoforms although this effect was not significant. Sig1R antagonist BD1047 in combination with cocaine decreased MAP-2 in uninfected tissues (Supplementary Fig.8a & 8b). On the other hand, MAP-2 isoforms A and B expression levels remained unchanged after treatments (Supplementary Fig.8c & 8d).

Unfortunately, we cannot conclude if BD1047 alone had an effect on MAP-2 expression due to insufficient tissues. Raw gels of respective blots for each of the proteins tested are included at the end of the Supplementary data file.

### BD1047 Prior to Cocaine Reduces CATB, Cleaved-Caspase-3, and p24 Antigen, but could not Recover Synaptophysin Expression on HIVE Mice Tissues

**Fig. 6:**
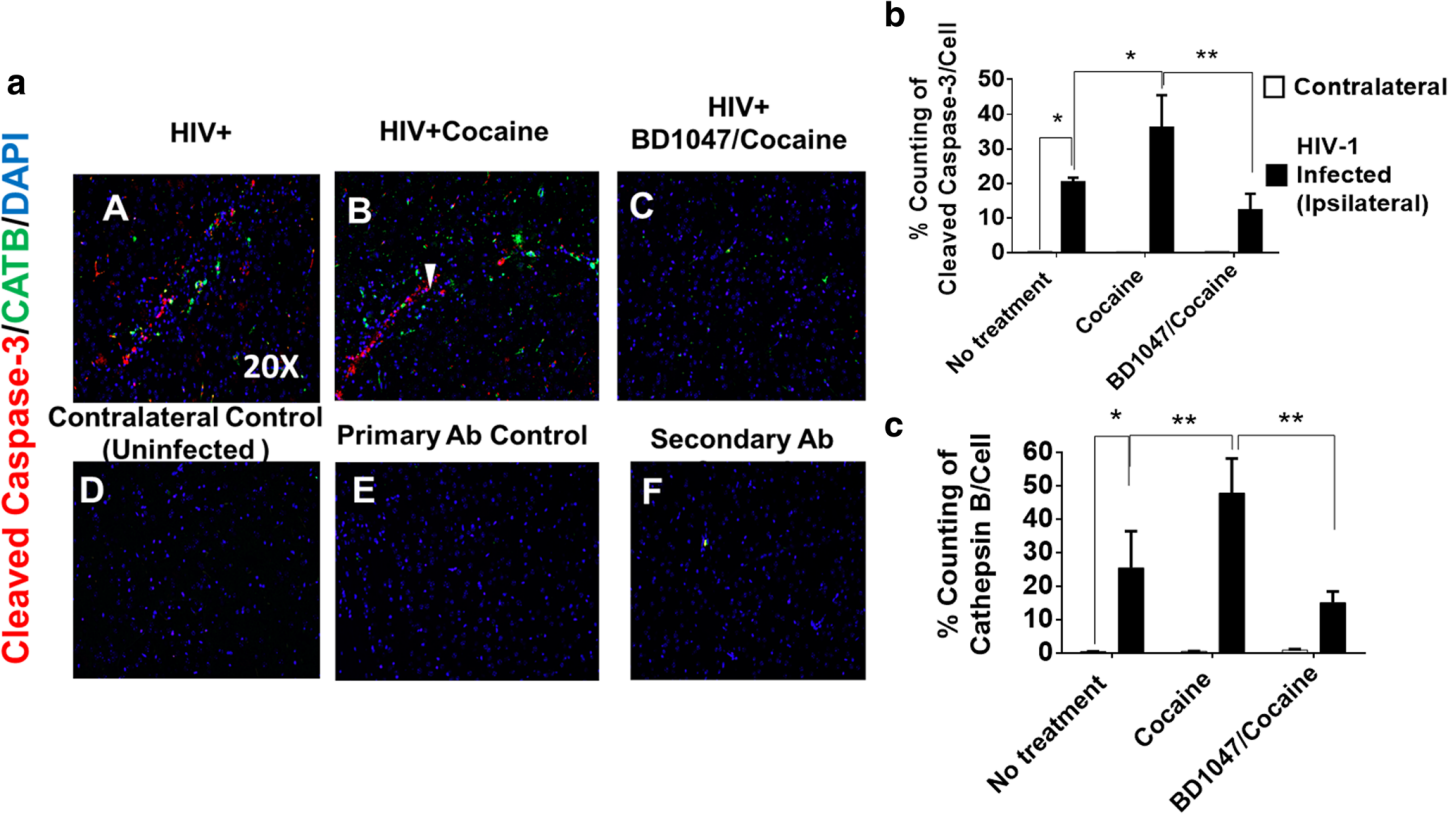
Immunostaining of Cleaved Caspase-3 and Cathepsin B (CATB) from the *Striatum* of HIV Encephalitis Mouse Model (HIVE). (**a**) Staining is from three mice (*n* = 3) with three randomized photos at 20X from ipsilateral (line of injection) and contralateral regions. Different treatments are depicted from (A-F). Contralateral controls are depicted as uninfected controls in this mouse model. Primary and secondary antibodies controls were used for each assay (**b**) Quantification of Immunostaining of Cleaved Caspase-3 (red staining) and Cathepsin B (CATB) (green staining) from the *Striatum* of HIV Encephalitis Mouse Model (HIVE). Quantification is done from three mice (*n* = 3) with three randomized photos at 20X. (**c**) Results are reported as (upper graph) single cleaved-caspase-3 counting/ DAPI (cell) for cleaved-caspase-3 and (**c**) for cathepsin B**.** Statistical Analyses were done with Two-Way ANOVA using the Tukey’s Post-Hoc test and Multiple Comparison Test (*p ≤ 0.05, **p ≤ 0.01)

**Fig. 7:**
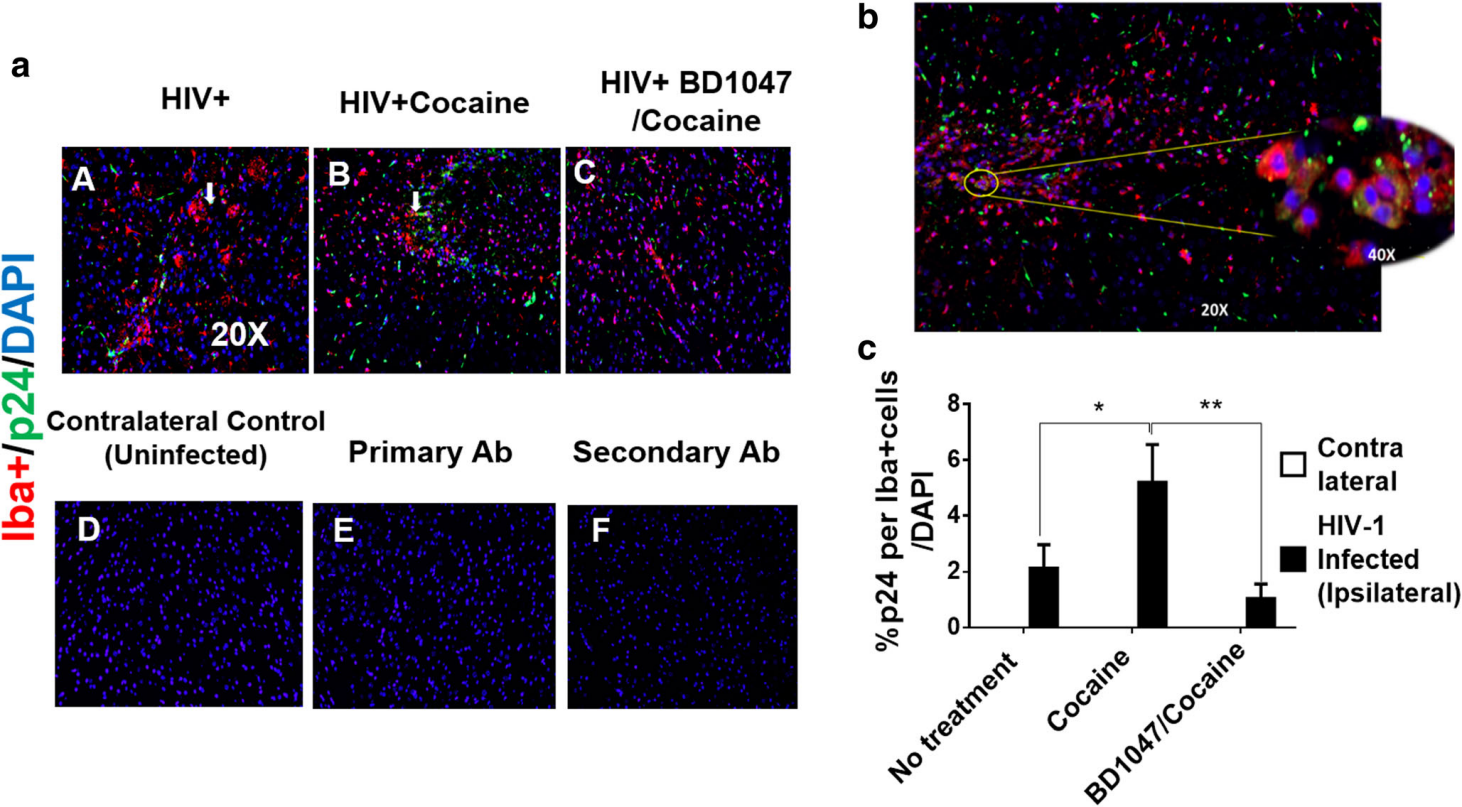
Immunostaining of Ionized calcium binding protein (Iba+) and p24 HIV-1 capsid protein from the *Striatum* of HIV Encephalitis Mouse Model (HIVE). (**a**) Staining is from three mice (n = 3) with three randomized photos at 20X from ipsilateral (line of injection) and contralateral regions. Different treatments are depicted from (A-F). Contralateral controls are depicted as uninfected controls in this mouse model. Primary and secondary antibodies controls were used for each assay. (**b**) Detail of p24 per Iba + (macrophages) colocalization in HIVE mice tissues at 20X and 40X. **c** Quantification of Immunostaining of Iba + (red staining) and p24 (green staining) from the *Striatum* of HIV Encephalitis Mouse Model (HIVE). Quantification is done from three mice (*n* = 3) with three randomized photos at 20X. Results are reported as percentage (%) of p24 colocalized per Iba+/ DAPI. Statistical Analyses were done with Two-Way ANOVA using the Tukey’s Post-Hoc test and Multiple Comparison Test (*p ≤ 0.05, **p ≤ 0.01)

To determine if Sig1R antagonist administered prior to cocaine was able to reduce HIV encephalitis neuropathology in mice, several markers for neuronal damage and apoptosis were used in paraffin-embedded tissues. Results demonstrate that cleaved-caspase-3 expression increased in HIV-infected tissues of cocaine treated mice (*p ≤ 0.05) but decreases with BD1047 treatment (**p ≤ 0.01) (Fig. 6a and b). HIV-infected tissues also showed increased CATB expression with HIV-infection and cocaine (*p ≤ 0.05) and this effect was abolished in those mice pretreated with BD1047 prior to cocaine as compared to the cocaine treated group (**p ≤ 0.01) (Fig. 6c). Treatment with BD1047 prior to cocaine also decreased HIV infection in macrophages as demonstrated by decreased p24 expression (Fig. 7). These results demonstrate that while cocaine increases each of these neurodegenerative markers in mice infected tissues, Sig1R antagonist (BD1047) prior to cocaine can reduce infection, CATB expression, and apoptosis. On the other hand, no changes were seen in synaptophysin (major synaptic vesicle protein p38) expression, indicating that these treatments do not show detrimental effects on neuro-synapses nor in synaptic transmission events with this marker and other parameters need to be considered in our studies (Supplementary Fig. 9).

We also stained for glial acid fibrillary protein (GFAP), present in astrocytes, on HIV-infected mice tissues. Results show an increased expression of GFAP on infected tissues with cocaine treatment (*p ≤ 0.05) compared to infected tissue with no treatments. BD1047 treatment prior to cocaine further reduced GFAP positive cells as compared to infected cocaine treated tissues (*p ≤ 0.05) (Supplementary Fig. 10).

### CATB and Sig1R Expression Increase in the Anterior *Basal Ganglia* of *Post-mortem* Human Brains of Seropositive Cocaine Users with HAND

**Fig. 8:**
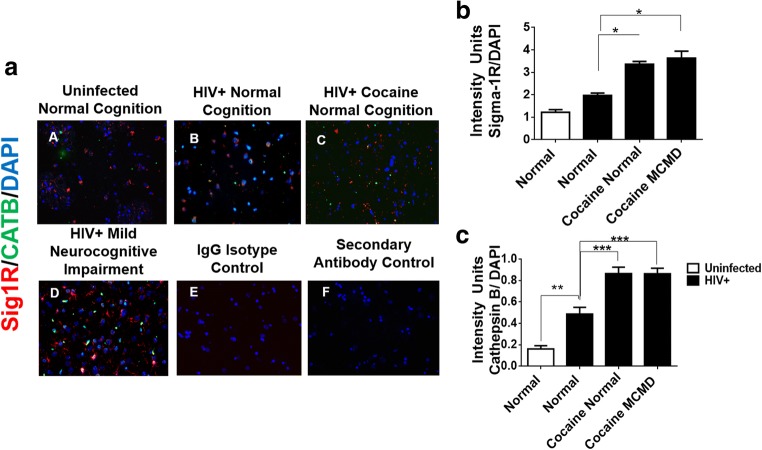
Immunohistochemistry of post-mortem human paraffin embedded- basal ganglia brain tissues. (**a**) Staining was performed using CATB (1:100) and Sig1R (1:200) as primary antibodies and DAPI staining for nucleus. (**b**) Quantification of Sig1R receptor relative intensity in tissues as normalized to DAPI. (**c**) Quantification of CATB relative intensity in tissues as normalized to DAPI**.** Immunohistochemistry staining colors are: DAPI (blue), sigma-1 receptor (red) and CATB (green) (*p ≤ 0.05, **p ≤ 0.01, ***p ≤ 0.001) (n = 3). Statistical Analyses were done with Two-Way ANOVA using the Tukey’s Post-Hoc test and Multiple Comparison Test

To confirm the association between the expression of Sig1R and CATB seen in HIVE mice, *post-mortem* human brain tissues from HIV seropositive patients who were cocaine users (provided by the National Neuro AIDS Tissue Consortia) were analyzed by immunohistochemistry. All the viral-immune and therapy parameters of the study patients are depicted (Supplementary Table 1). We used all the patients for the analyses. Two of the patients with normal cognition did reported non-drug use prior to death but did not have urine toxicology test performed. These patients had thus an unknown status of cocaine use. A significant increase in Sig1R was observed in individuals who were cocaine users either with normal cognition or with some forms of mild neurocognitive impairment compared to non-drug users (*p ≤ 0.05) (Fig. 8a). No significant differences in Sig1R expression were detected between HIV-seropositive compared to HIV-seronegative individuals (Fig. 8b). CATB expression was also analyzed in these subjects. An increased expression of CATB was found in HIV-seropositive compared to HIV-seronegative individuals as well as in cocaine users compared to non-users (Fig. 8c). Individuals with normal cognition who were HIV seropositive had greater expression of CATB when compared with the HIV negative individuals (**p ≤ 0.01). HIV seropositive individuals with normal cognition that used cocaine had greater expression of this protein when compared with non-cocaine users (***p ≤ 0.001). In addition, HIV-seropositive individuals who were cocaine users with some forms of mild neurocognitive impairments (MCMD) had greater expression of CATB when compared with HIV seropositive individuals with normal cognition (***p ≤ 0.001) (Fig. 8c).

Our results confirm previous studies that show that HIV infection and cocaine use have a direct effect on increasing the expression of CATB (Zenón et al. 2014). Although in this study we could not compare the effect of HAND on CATB expression due to limited number of samples, previous studies confirmed an increased CATB levels with HAND (Cantres-Rosario et al. 2015).

## Discussion

Cocaine abuse remains one of the main hurdles of HIV-1 eradication and treatment (Larrat and Zierler 1993). Not only this substance increases viral progression but also potentiates HIV-1 associated neurocognitive disorders (HAND) severity in patients (Dahal et al. 2015). Biologically, cocaine enhances HIV-1 replication in several immunological cells including peripheral blood monocytes and macrophages (Peterson et al. 1991). We have reported in previous studies that cocaine increases HIV-1 replication and CATB secretion in MDM that exacerbates neuronal apoptosis (Zenón et al. 2014). This previous study also examined human brain hippocampal tissue and observed an increase in CATB secretion in HIV positive women with a history of cocaine use. Our results confirmed these previous studies of increased CATB in *post-mortem* human brain striatal tissue with a history of cocaine use as compared to non-drug users. In addition, we measured CATB expression and assessed neuronal function in the HIVE animal model. Results obtained from mice striatal brain tissue confirmed increased expression of CATB along the line of injection of human MDM by immunohistochemistry. However, CATB was not different in the whole striatal lysates by Western blots (Supplementary Table 2). Differences between techniques include measurements of other areas of the mouse brain by Western Blots when compared to expression along the line of human-MDM injection by immunohistochemistry. It will be relevant to pursue further studies using humanized HIV mice.

The main purpose of the present study was to determine if cocaine increases HIV-infection and CATB secretion by interacting with the Sig1R. This was accomplished by using the specific Sig 1R agonist BD1047 and the sigma 1 receptor agonist PRE-084 in our MDM in vitro studies and with the HIVE in vivo model. In examining Sig1R expression in vitro using human monocyte-derived macrophages or MDM, intracellular levels of this receptor were found unaltered after cocaine exposure and after pretreatment with BD1047 antagonist. Our in vivo studies with the HIVE mice model yield similar results. However, human *striatal* tissues from HIV-seropositive patients that tested positive for cocaine show an increase in Sig1R immunoreactivity compared to seronegative non-cocaine controls. Differences in Sig1R expression in our experimental models might be due to diverse immunological milieu and on protein abundance in the region of interest. Results also indicate that mice treated with cocaine show increased HIV-1 p24 immunostaining in the striatum compared to mice that did not receive cocaine, similar to our in vitro findings using MDM. In the same manner, cocaine increased total apoptosis (cleaved caspase-3) and induced astrocytosis in vivo as determined by increased GFAP expression and immunostaining in HIVE tissues. These detrimental effects of HIV and cocaine have been described in studies with HIV patients with neurocognitive impairment (Larrat and Zierler 1993). Increased neuronal apoptosis after exposure to HIV-infected cocaine treated MDM supernatants were quantified by DNA damage (TUNEL), while increased total caspase-3 dependent apoptosis immunostaining was observed for HIVE mice studies. Although no direct correlation of Sig1R modulation by cocaine and CATB has been described in literature, previous findings suggested to us that this substance-receptor interaction promotes calcium dysregulation and oxidative stress that might affect lysosomal integrity indirectly (Hayashi and Su 2007; Boya and Kroemer 2008). Modulation of Sig1R by BD1047 antagonist prior to cocaine has been demonstrated to reduce most of these effects in monocytes, macrophages, and HIV-1 levels in microglia (Yao et al. 2010; Gekker et al. 2006). In this publication the authors show that cocaine’s occupancy on Sig1R of mice brain are almost 31% when using doses lower than 15 mg/kg/weight (equivalent to 31.6 μmol). Therefore, we expected that a 15 mg/kg/weight cocaine dose saturate over 31% of the Sig1R in the mouse brain.

Our results indicate that BD1047 obliterates cocaine action in HIV-1 infection, CATB levels and neuronal apoptosis when compared to cocaine treated MDM without the antagonist. In HIVE mice *striatal* tissues, treatment of BD1047 prior to cocaine decreased p24 per Iba + (macrophages and microglia) immunostaining. It is important to mention that while in MDM p24 and CATB levels were measured extracellularly, in mice tissues these proteins were measured intracellularly in a differential environmental milieu. Conversely, BD1047 treatment of MDM supernatants prior to cocaine reduced apoptosis of neurons that was specific to an increase in CATB as apoptosis was also reduced with CATB antibodies. Nevertheless, it is important to note that MDM supernatants may contain other factors that contribute to neuronal apoptosis such as HIV-1 viral proteins and reactive oxygen species among others. In mice tissues, less apoptosis was seen on infected striatal brain tissue treated with BD1047 prior to cocaine in relation to their counterparts treated with cocaine. Other hallmarks of HIV related neuropathology such as increased glial fibrillary acidic protein (GFAP), characteristic of astrocytosis, were reduced by these treatments while other markers such as synaptophysin, characteristic of synaptic dysfunction, were unchanged. However, other markers such as microtubule associated protein-2 (MAP-2) isoforms C &D were reduced with BD1047 and cocaine treatments in uninfected tissues. These results might indicate that while juvenile isoforms of MAP-2 (C& D) were reduced with infection and in uninfected tissues treated with BD1047 and cocaine, mature isoforms of this protein (A&B) were not affected by these treatments. Thus, earlier stages rather than later stages of neurogenesis might be affected after each of the treatments, but further experiments are required to confirm this observation. Yet, our mice model complied with all classical HIV-1 related neuropathology markers as confirmed by our studies and from Persidsky and Gendelman 1997. In this study we have not found differences in Iba-1 expression among uninfected and infected mice tissues. As the Iba-1 recognize both human and mouse antigens, this phenomenon can be explained by the fact that a fixed number of human macrophages are injected into the *striatum* and that mice microglia might not be susceptible to activation as seen in human microglia.In this study, we also explored the possibility of an effect of a Sig1R agonist (namely PRE-084) administered prior to cocaine on HIV-1 infection and CATB levels in MDM. We hypothesized that since cocaine is described as an agonist of Sig1R, PRE-084 along with cocaine will further potentiate cathepsin secretion and increase HIV-1 levels in MDM. Our results did not confirm our hypothesis (Supplementary Table 2). Treatment of infected MDM with PRE-084 prior to cocaine had no effect on HIV-1 infection or CATB secretion, thus in vivo studies with the HIVE mouse model were not carried out. Several studies have demonstrated that PRE-084 can exert antioxidant, anti-calcium, and anti-inflammatory responses within the cell highlighting the beneficial effects of Sig1R activation in lysosomal integrity (Allahtavakoli and Jarrott 2011; Nguyen et al. 2014; Katnik et al. 2006) contrary to what we observed in this study. It is possible that cocaine’s effect on oxidative stress or calcium imbalance is greater than the ability of PRE-084 of counteracting this effect. A summary of main findings in MDM, HIVE mice, and human brain tissues in the presence and absence of cocaine is presented in Supplementary Table 2.

## Concluding Remarks

Our findings demonstrate that the Sig1R antagonist (BD1047) reduces the effect of cocaine in HIV-1 infection in vitro and in vivo. BD1047 reduces CATB secretion of MDM cultures when administered prior to cocaine, and of CATB immunoreactivity in striatal neurons of HIVE mice. Neuronal apoptosis is also reduced after BD1047 treatment prior to cocaine and in *striatal* mice tissues. Other hallmarks of HIV-1 neuropathology are also reduced after BD1047 pretreatment prior to cocaine such as caspase 3, MAP-2, and DNA damage. These results demonstrate that pharmacological modulation of Sig1R through BD1047 is capable of reducing the effect of cocaine in HIV-infection, CATB, and HIV-related neuropathology markers in vitro and in vivo*.* However, differences on CATB and Sig1R expression in MDM, mice, and human tissues suggest that other factors, such as: cytokines, hormones, and cell interactions, may be involved as well. Future studies using other animal models such as the humanized HIV mice and the SIV monkey model may provide clarification.

On the other hand, Sig1R agonist (PRE-084) prior to cocaine had no effect on cathepsin B or HIV-1 infection. However, PRE-084 might be a suitable candidate for future HIV-1 studies that do not involve cocaine since it decreased CATB secretion in MDM in absence of cocaine.

We are aware of the study limitations and the long road before considering implementation of BD1047 as a therapeutic approach against the action of cocaine in HIV-1 seropositive patients. Nonetheless, Sig1R antagonist BD1047 has a potential for use along with antioxidants such as dimethyl fumarate (Rosario-Rodríguez et al. 2018) for the reduction of CATB related neuropathology in HIV-1 seropositive patients. Therefore, Sig1R antagonist BD1047 warrants further studies that may uncover its therapeutic potential.

## Electronic Supplementary Material

ESM 1 (PDF 1520 kb)
