# Supplementary material for: Sigma-1 Receptor Antagonist (BD1047) Decreases Cathepsin B Secretion in HIV-Infected Macrophages Exposed to Cocaine
Source: J Neuroimmune Pharmacol. 2018 Oct 10;14(2):226–40. doi: 10.1007/s11481-018-9807-4 (PMC6488453; doi:10.1007/s11481-018-9807-4)

Supplementary Figure 1: Sig1R antagonist BD1047 titration at 11 days post-infection. Dose dependent curve of BD1047 from 0.001 µM to 10 µM to concentration. p24 levels (pg/mL) from 11 days post-infection **(a)** and CATB secretion (ng/mL) at 11 days post-infection (dpi) **b).** Four donors were used (n=4). Data was analyzed by means of a Two way-ANOVA and Tukey's multiple comparison test

(* p≤0.05, ** p≤0.01).

a

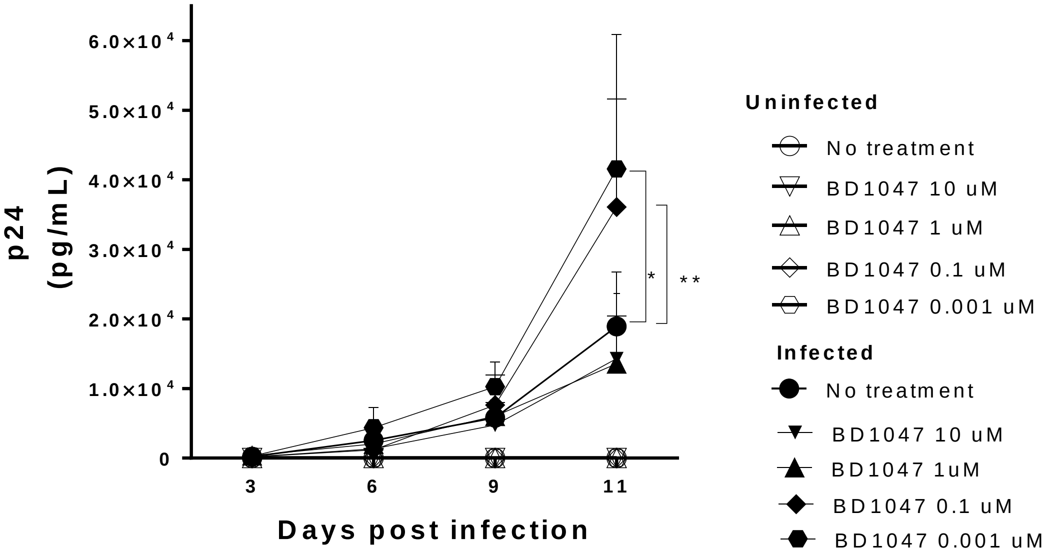

b

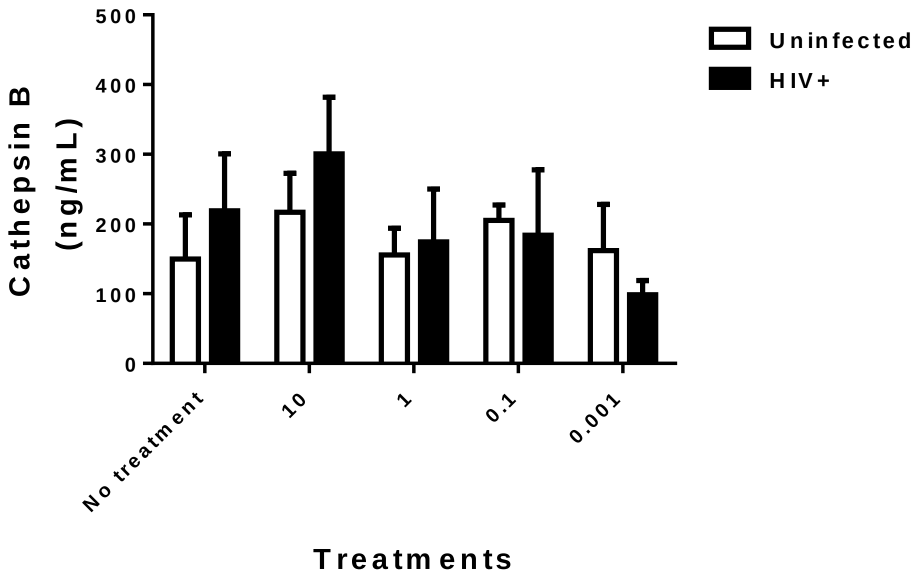

Supplementary Figure 2: Sig1R antagonist BD1047 titration at 11 days post-infection. Cell viability in presence of BD1047 at 0.01 to 10 µM was measured by means of MTT assay in absence of cocaine. Results represent percentage of cells viable of each of the individual treatments divided by their respective non-treated controls and multiplied by hundred (100) **(a).** BD1047 cell viability in presence of cocaine at 11 days post-infection. Sig1R antagonist BD1047 was titrated in presence and absence of cocaine to determine its effect in cell viability by means of an MTT assay **(b).** Statistics were done by means of a Two-Way ANOVA using multiple comparison mean test. Non- significant differences were seen between each of the treatments. Three donors are depicted (n=3)

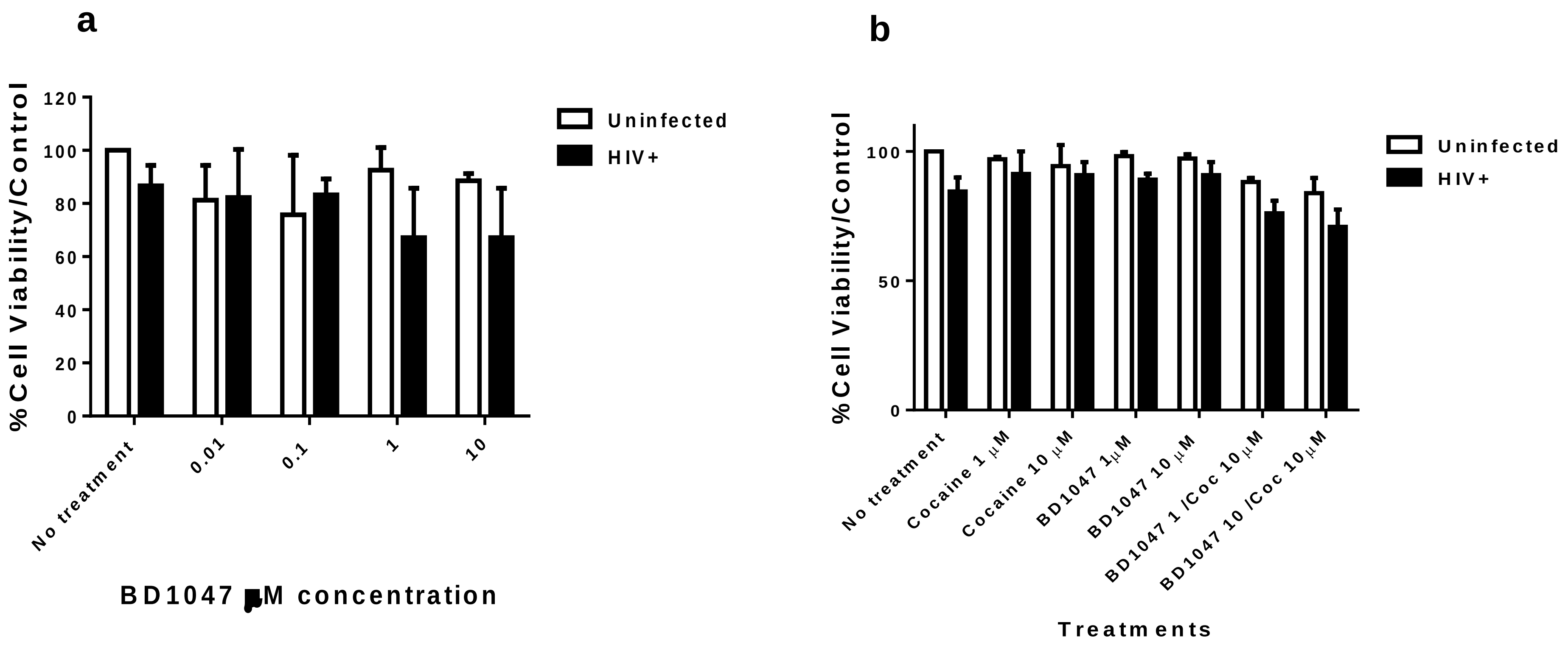

Supplementary Figure 3: (a) Predictive margins for Multiple Way ANOVA correlations HIV status, cocaine and BD1047 treatments in cathepsin B secretion as determined by Stata Program. Legend: 0=Absence, 1= Presence. **(b)** Predictive margins for Multiple Way ANOVA correlations HIV status, cocaine and BD1047 treatments in p24 levels in MDM as determined by Stata Program. Legend: 0=Absence, 1= Presence

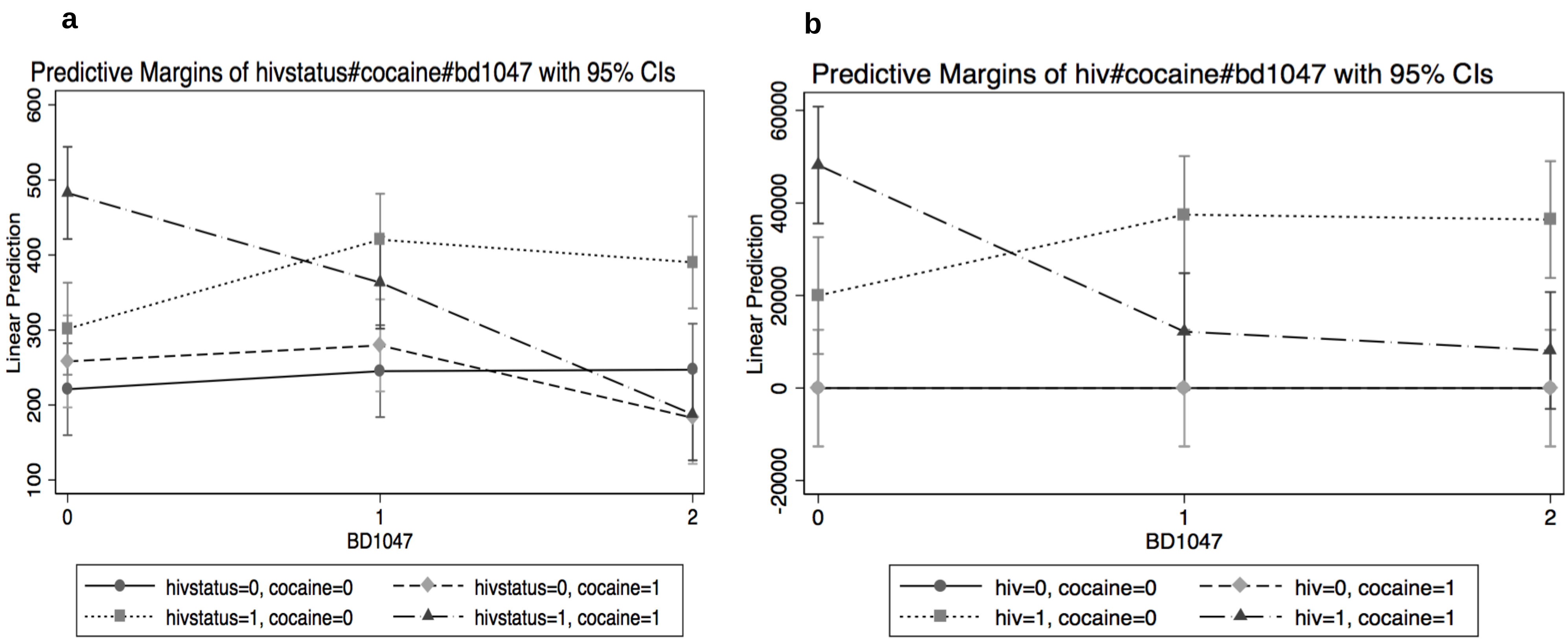

Supplementary 4: Sig1R agonist PRE084 titration at 11 days post-infection. p24 antigen levels (pg/mL) in presence of PRE084 at 0.0001 to 1 µM concentration was measured by means of an ELISA. p24 levels from 11 days post-infection **(a)** and CATB secretion (pg/mL) at 11 days post-infection **(b).** Data was analyzed by means of a Two way-ANOVA and a multiple comparison test. Four donors depicted (n=4). (*p≤0.05, ** p≤0.01, *** p≤0.001, ****p≤0.0001).

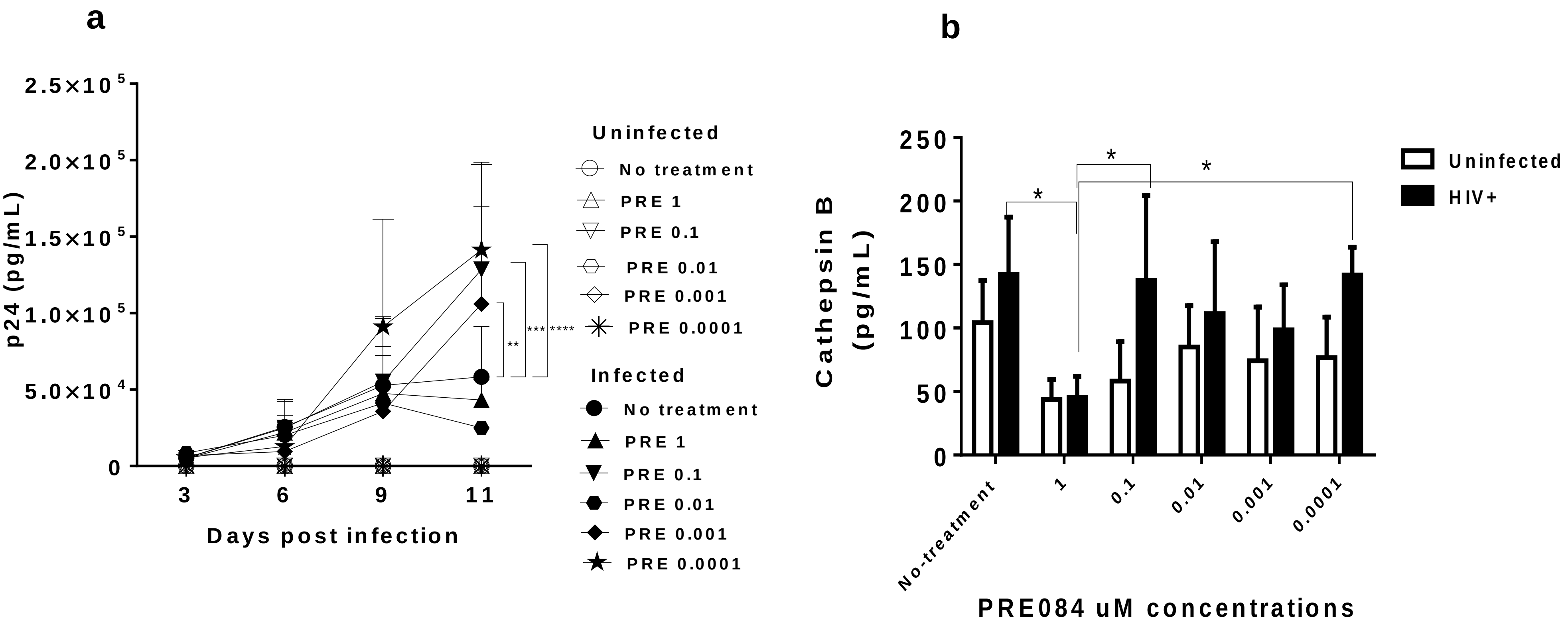

Supplementary Figure 5: Sig1R agonist dose curve titration at 11 days post-infection. Sig1R agonist PRE-084 was titrated to determine its effect in cell viability by means of an MTT assay. Results represent percentage of viable cells after each of the individual treatments divided by their respective non-treated controls and multiplied by hundred (100) **(a)**. Sig1R agonist dose curve titration at 11 days post-infection in presence of cocaine. Sig1R agonist PRE-084 was titrated in presence of cocaine to determine its effect in cell viability by means of an MTT assay. **(b).** Statistics were done by means of a Two-Way ANOVA using multiple comparison mean test. Non- significant differences were seen between each of the treatments. Three donors depicted (n=3).

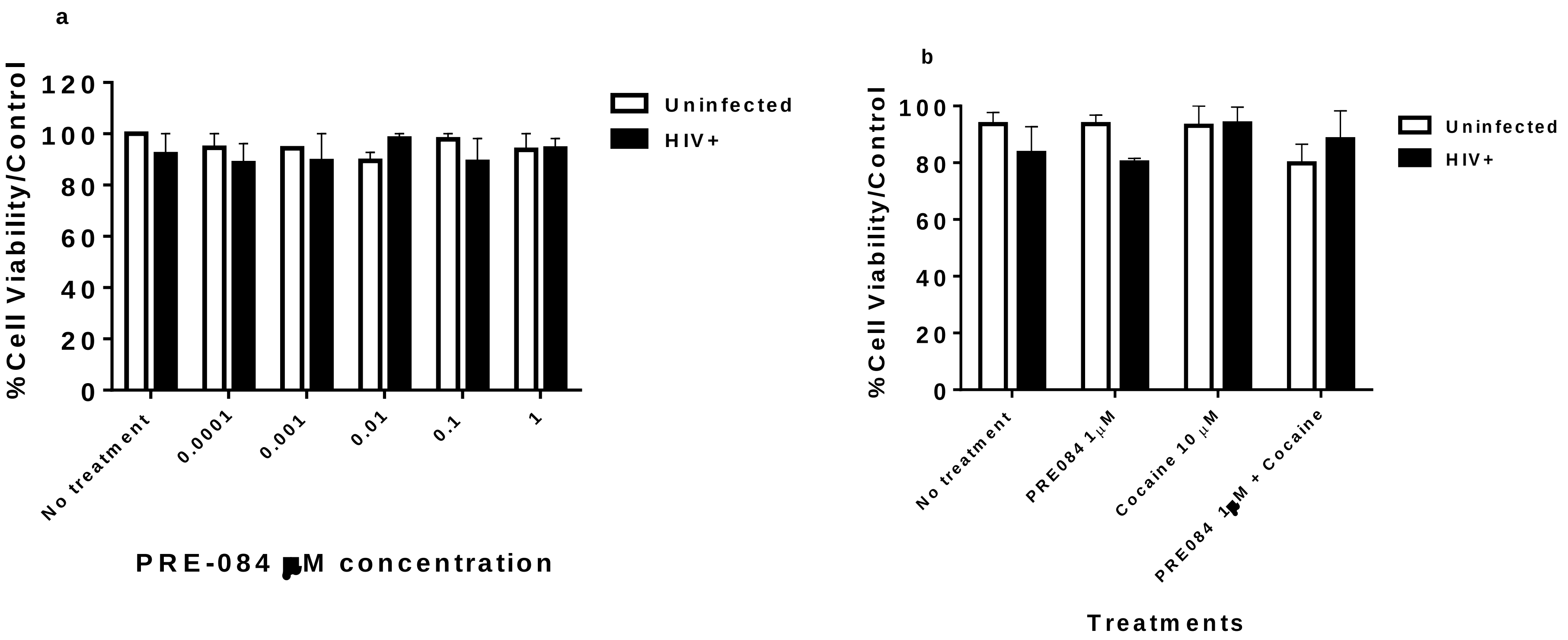

Supplementary Figure 6: Neuroblastoma Cell Lines (HTB-11) cell death (as measured by TUNEL) after treatment with MDM Uninfected Conditioned Media (MCM). Notice the lack of green fluorescence indicative of cell death. Infected controls and their related quantification are part of **Fig.6a & 6b.** Results representative from four donors (n=4)

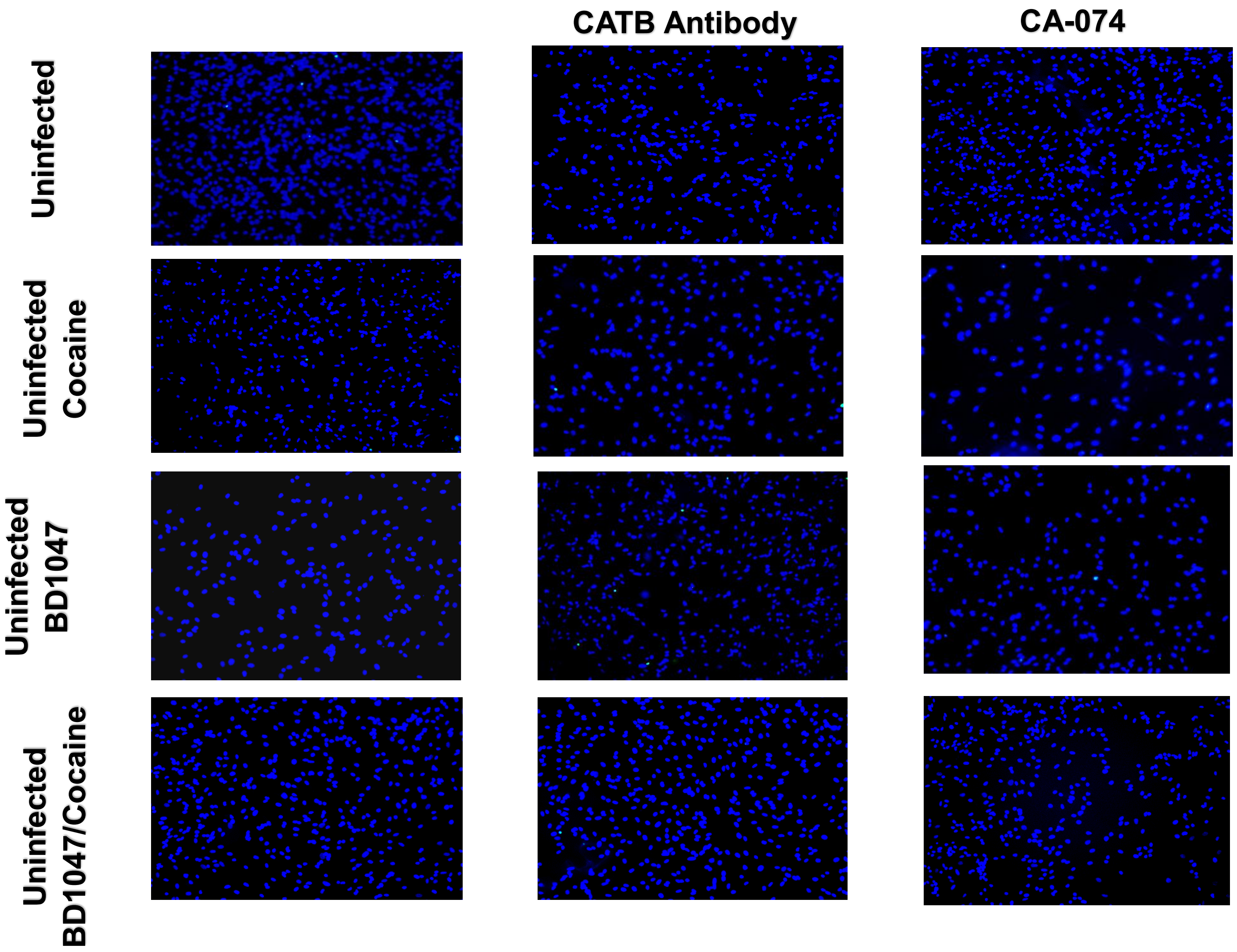

Supplementary Figure 7: Intracellular expression of sigma-1 receptor and cathepsin B in HIV-1 infected macrophages treated with cocaine as measured by Western Blot **(a)** and related densitometry**(c)** of sigma-1 receptor (Sig1R) intracellular expression and of **(b)** cathepsin B in HIV infected or uninfected macrophages not treated or treated with cocaine at 12 days post-infection and**(d)** its quantification **(**Statistical Analyses were done with Two-Way ANOVA using the Tukey's Post-Hoc test and Multiple Comparison Test).

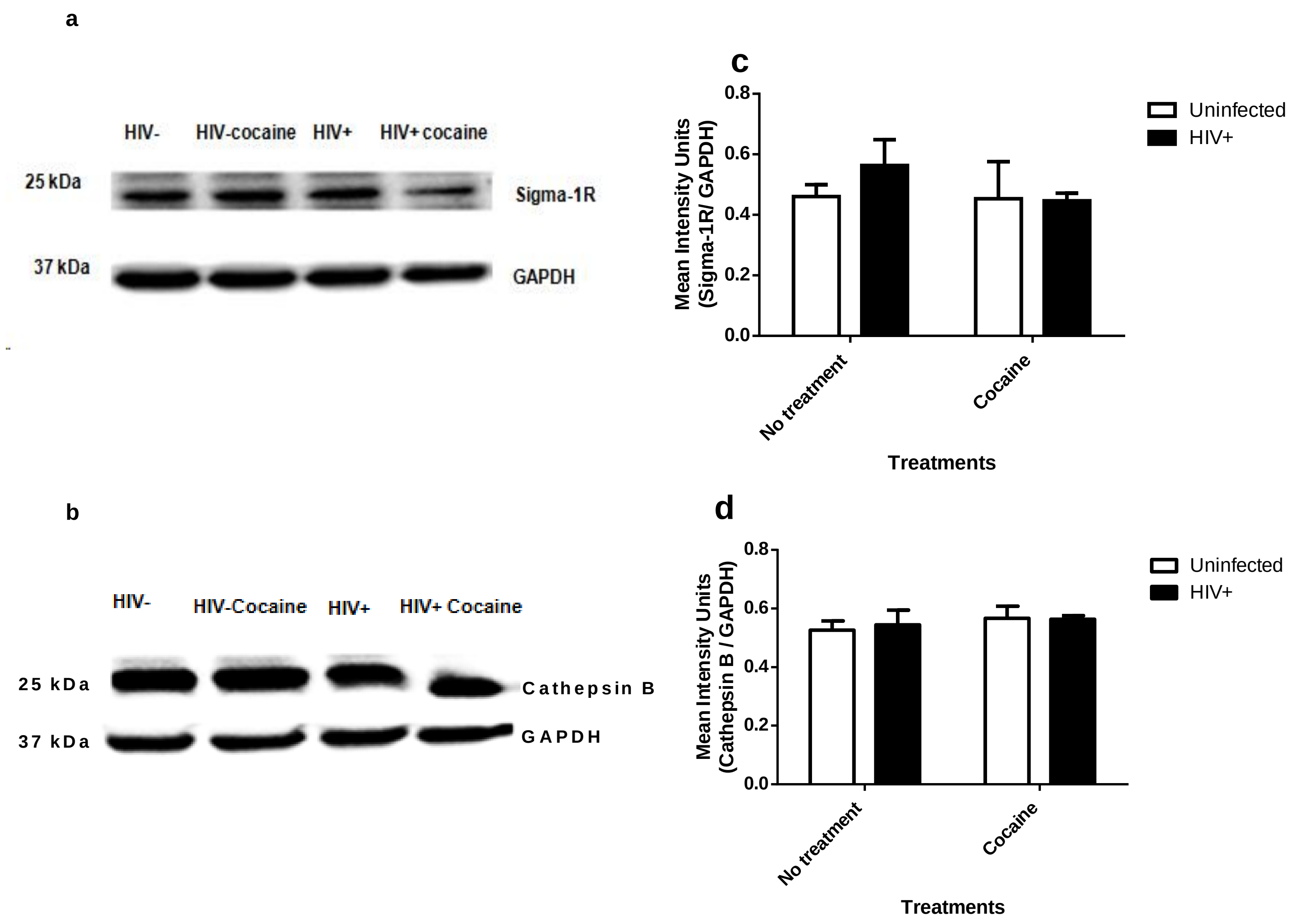

Supplementary Figure 8: Western Blot of microtubule associated protein-2 (MAP-2) isoforms expression in the *striatum* of HIVE mice. Blots of MAP-2 expression low molecular weight isoforms C & D after 14 days of injection of infected MDM into striatum and cocaine treatments or BD1047/cocaine treatments (*p≤0.05, **p≤0.01) (n=4 per group) **(a)** Blot of MAP-2 isoforms C & D and **(b)** normalization using GAPDH as an internal control. Uninfected mice show a higher expression of MAP-2 C& D isoforms than infected mice tissues. **(c)** Blot of MAP-2 high molecular weight isoforms A&B and **(d)** normalization with GAPDH. No differences in MAP-2 A&B isoforms were seen between groups.

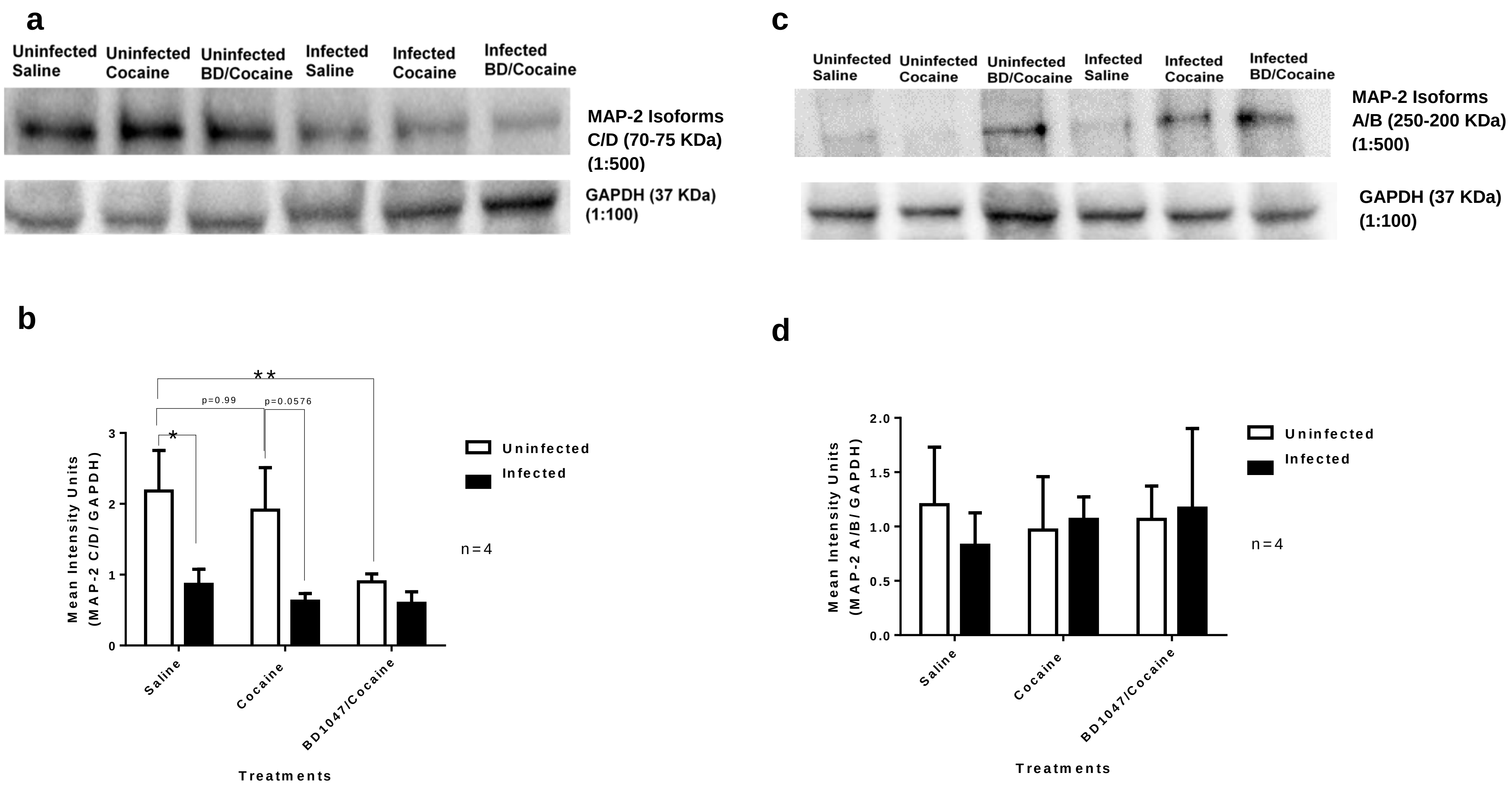

Supplementary Figure 9: Immunostaining of Synaptophysin and CATB from the *Striatum* of HIV Encephalitis Mouse Model (HIVE). Staining is from three mice (n=3) with three randomized photos at 20X from ipsilateral (line of injection) and contralateral regions. Different treatments are depicted from (A-C). Controls are depicted on panels (D-F) **(a).** Quantification of Immunostaining of synaptophysin **(red staining)** and CATB **(green staining)** from the *Striatum* of HIV Encephalitis Mouse Model (HIVE). Results are reported as single synaptophysin integrated density/ DAPI (cell) for synaptophysin as well as for (**b**) for CATB **(c).**

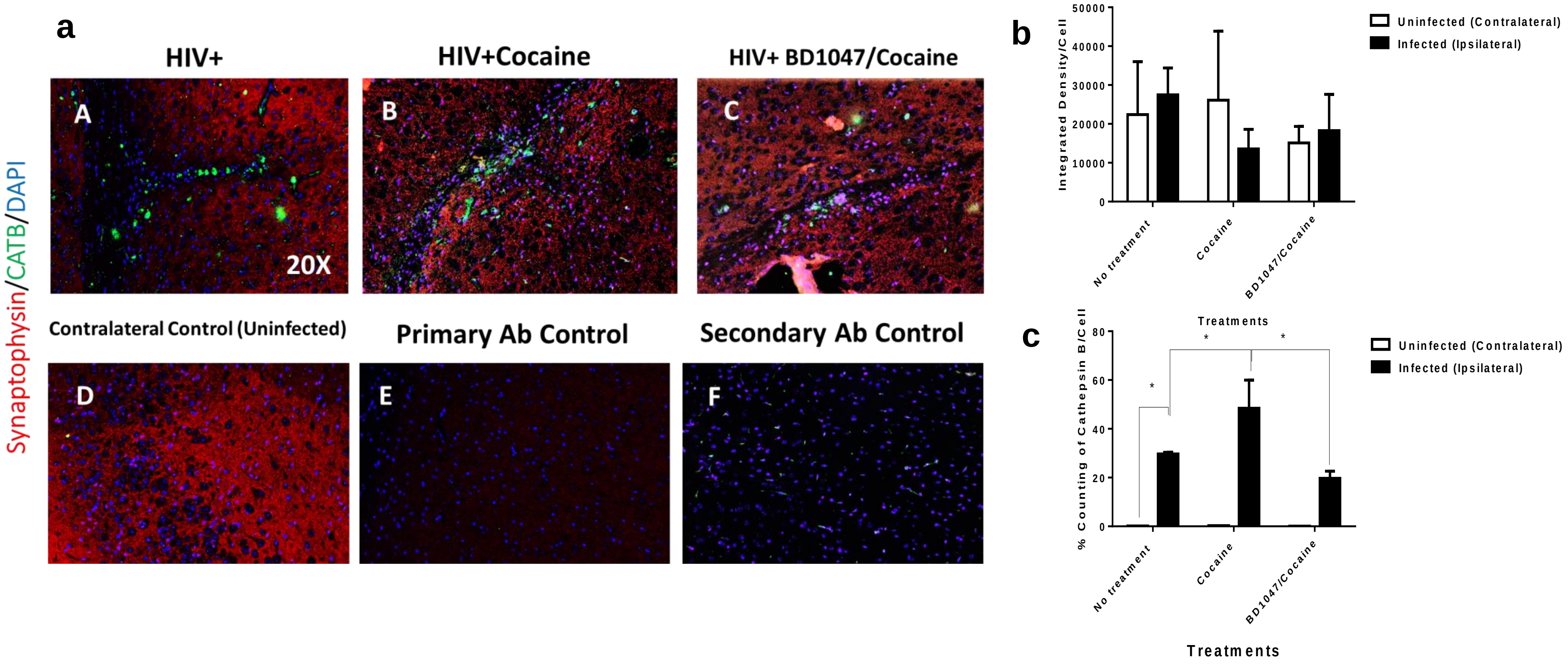

Supplementary Figure 10: (a) Immunostaining of Glial Acidic Fibrillary Protein (GFAP) from the *Striatum* of HIV Encephalitis Mouse Model (HIVE). Staining is from three mice (n=3) with three randomized photos at 20X from ipsilateral (line of injection) and contralateral regions. Different treatments are depicted from (A-C) Controls are depicted on panels (D-F)**. (b)** Quantification of Immunostaining of GFAP **(red staining)** from the *Striatum* of HIV Encephalitis Mouse Model (HIVE). Blue color (DAPI) stains nuclei of cells.

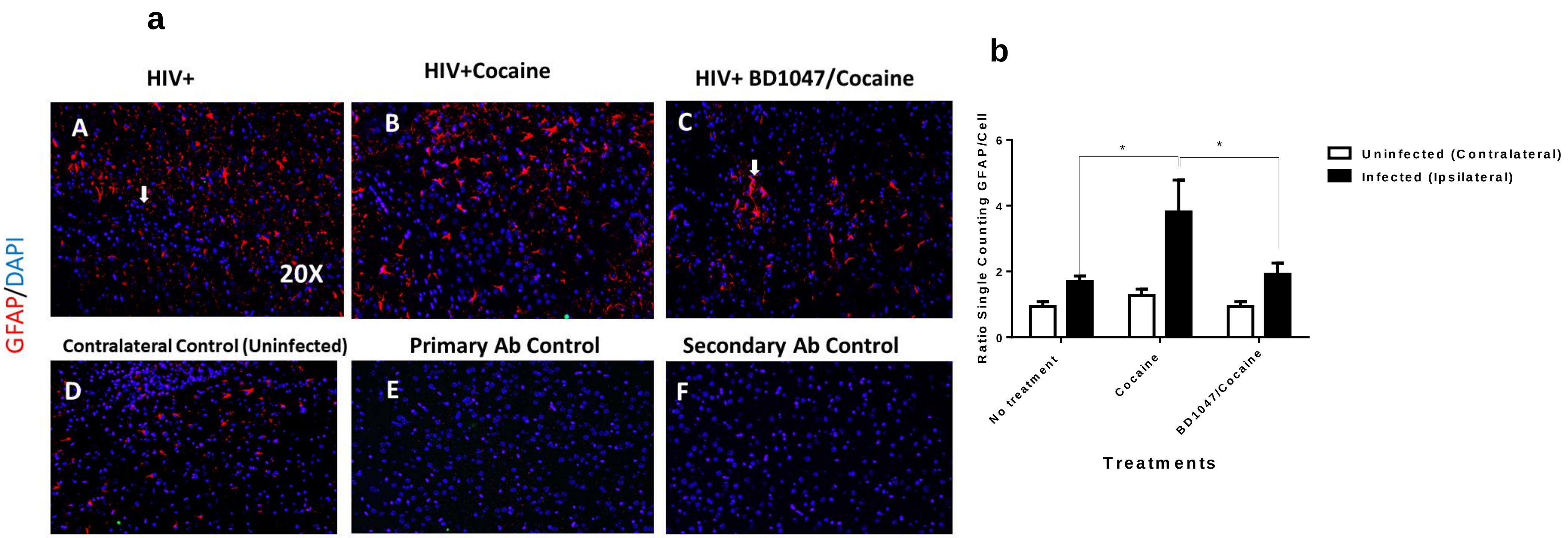

Supplementary Table 1. *Post-mortem* Brain Tissues and patient status received from the National Tissue Consortium (NNTC) – Brain tissues from patients are from 10-20 µM thick section slides of anterior basal ganglia.

Case ID [1]	Age[2]	CD4[3]	CD8[4]	Plasma VL[5]	CSF VL[6]	HIV infected	Cocaine Abuse (Urine Toxicology)	Self-report of past abuse	Antiretroviral drugs pre-mortem	Neurocognitive Functions[7]	Neurocognitive Status [7]
**UCLA 5007**	37	92	1155	750000	NA	Yes	Positive	None	DDI, EFV, KTA, NVP, TFV, TZV	2	Possible mild neurocognitive disorder (MCMD)
**UCLA 2005**	43	66	506	750000	249	Yes	Positive	Alcohol, Cannabis, Hallucinogen, Opiate, Stimulants	DDI, KTA, RTV, TFV	1	Neuropsychological impairment; does not meet criteria for syndromic disorder
**UCLA 4148**	52	160	NA	700	NA	Yes	Negative	None	3TC, ATR, D4T, FTV, NFV DRV, EPZ, KTA, RTV, TFV, TRU, RTV	2	Possible MCMD
**UCLA 3030**	59	1191	190	400	NA	No	Unknown	None	No data available	0	Normal cognition
**UCLA 3039**	70	NA	NA	NA	NA	No	Unknown	None	No data available	0	Normal Cognition
**UCLA 4049**	NA	NA	NA	NA	NA	Yes	Negative	None	APV, D4T, DDI, NVP	0	Normal Cognition
**UCLA 5007**	37	91	1155	750000	NA	Yes	Positive	None	DDI, EFV, KTA, NVP, TFV, TZV	2	Possible MCMCD
**UCLA 5016**	NA	NA	NA	NA	NA	Yes	Positive	None	ATV, CBV, DDI, NFV, NVP, TFV, ZDV, EFV, RTV, T20	1	Neuropsychological impairment; does not meet criteria for syndromic disorder
**UCLA 4028**	NA	NA	NA	NA	NA	Yes	Yes	Alcohol, Cannabis, Cocaine	3TC, D4T, NVP	4	Possible HAD

Table 1. Patients were stratified based on NA= not available. Patient identification (1), age (2), and different immune parameters including C4 and CD8 nadir (3-4 respectively), plasma and CSF viral load in copies per mL (5-6). Neurocognitive status (7) 1= Neurocognitively normal, 2=Possible mild neurocognitive impairment (MCMD), 3= Probable MCMD, 4=Possible HIV associated dementia. Other drugs of use at *pre-mortem* are based on clinical visits self-reported by patients as past-current abuse. These provided here are thought to be all reported under past-present use within the study. In terms of medications, antiretrovirals reported are based on complete antiretroviral regime (which includes those from all patients visits to the ones consumed at the time of death). Antiviral medication acronyms: 3TC; lamivudine (Epivir), ABC= abacavir (Ziagen), ADV; adefovir dipivoxil (Preveon; Hepsera,, APV; amprenavir (Agenerase), ATC; apricitabine, ATR; efavirenz 600mg + emtricitabine 200mg + tenofivir DF 300mg (Atripla), ATV; atazanavir (Reyataz), BIK; bictegravir 50mg + emtricitabine 200mg + tenofovir alafenamide 25mg (Biktarvy), BSD; Blinded Study Drug or Otherwise Unknown, CBV; zidovudine + lamivudine (Combivir), COM; emtricitabine 200mg + rilpivirine 25mg + tenofovir DF 300mg (Complera), CPV; capravirine, d4T; stavudine (Zerit), ddC; zalcitabine (Hivid), ddI; didanosine (Videx), DLV; delaviridine (Rescriptor), DSC; Emtricitabine (FTC) + Tenofovir Alafenamide (TAF) (Descovy), DTG; dolutegravir (Tivicay), EFV; efavirenz (Sustiva), EMV; MKC-422, emivirine (Coactinon), EPZ; ziagen + epivir (Epzicom), EVG; Elvitegravir, EVO; atazanavir 300mg + cobicistat 150mg (Evotaz), FPV; fosamprenavir (Lexiva), FTC; emtricitabine (Coviracil; Emtriva), GEN; emtricitabine + tenofovir alafenamide (TAF) + elvitegravir + cobicistat (Genvoya), HU; hydroxyurea, IDV; indinavir (Crixivan), JUL; dolutegravir 50mg + rilpivirine 25mg (Juluca), LPV/RTV; lopinavir/ritonavir (Kaletra) MVC; maraviroc (Selzentry), NFV; nelfinavir (Viracept), None, NVP; nevirapine (Viramune), PRE; darunavir 800mg + cobicistat 150mg (Prezcobix),QUA; TRU + elvitegravir + cobicistat (Stribild formally Quad), RFT; rilpivirine (RPV) + emtricitabine (FTC) + tenofovir alafenamide (TAF) (Odefsey), RGV; raltegravir (Isentress), RMN; Salk vaccine (Remune), RPV; rilpivirine (Edurant), RTV; ritonavir (Norvir), SCH; Schering #138, SQV2 or FTV; saquinavir-sgc (Fortovase), SQV; saquinavir (Invirase), T20; enfuvirtide; pentafuside (Fuzeon), TFV, PMPA; tenofivir DF (Viread), DRV; TMC-114, darunavir (Prezista), TMC; TMC-125, etravirine (Intelence), TMQ; abacavir 600mg + dolutegravir 50mg + lamivudine 300mg (Triume), TPV; tipranavir (Aptivus), TRU; emtricitabine + tenofovir (Truvada), TZV; AZT + 3TC + abacavir; zidovudine + lamivudine + abacavir (Trizivir), VCV; vicriviroc, ZDV; AZT, zidovudine (Retrovir), ZTV; AR-177 (Zintevir).

Supplementary Figure Table 2: (a) Key findings of *in vitro* and *in vivo models* after BD1047 treatment prior to cocaine in terms of HIV-1 infection, CATB, Sig1R and apoptosis markers (b) with PRE-084 treatment prior to cocaine in MDM.

a

Model	Treatments	*HIV infection	**Cathepsin B	***Apoptosis	****Sig1R	Comments
				In Vitro		
**MDM** 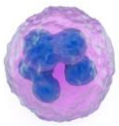	**HIV+**	↑	↑	NT	—	* p24 levels and ** CATB were tested from MDM supernatants by ELISA 11dpi<br><br>****Sig1R levels were tested from MDM lysates by Western Blots 12 dpi
	**HIV+ Antagonist**	—	—	NT	NT	
	**HIV+ Cocaine**	↑	↑	NT	—	
	**HIV+Antagonist/Cocaine**	↓	↓	NT	NT	
**Neurons** 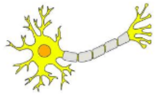	**HIV+**	NT	NT	↑	NT	***Apoptosis of neurons (DNA damage) were tested after exposure to MDM conditioned media (MCM) from 12 dpi by TUNEL Fluorescent Assays
	**HIV+ Antagonist**	NT	NT	—	NT	
	**HIV+ Cocaine**	NT	NT	↑	NT	
	**HIV+Antagonist/Cocaine**	NT	NT	↓	NT	

			In Vivo			
**HIVE Mice** 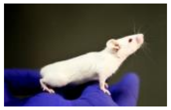	**HIV+**	↑ (IHC)	↑ (IHC, WB)	↑ (IHC)	— (WB)	*, **, ***- Tested by IHC or WB of HIVE mice striatum tissues
	**HIV+ Antagonist**	**NT**	**NT**	**NT**	— (WB)	
	**HIV+ Cocaine**	↑ (IHC)	↑ (IHC) — (WB)	↑ (IHC)	— (WB)	*Different mice used for IHC and WB
	**HIV+Antagonist/Cocaine**	↓ (IHC)	↓ (IHC, WB)	↓ (IHC)	— (WB)	*, **, ***- Tested by IHC or WB of HIVE mice striatum tissues
**Human Brain Tissues** 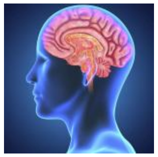	**HIV+ Normal Cognition**	**Viral levels at Table 1**	↑ (IHC)	**NT**	↑ (IHC)	
	**HIV+ Cocaine Normal Cognition**	**Viral levels at Table 1**	↑ (IHC)	**NT**	↑ (IHC)	
	**HIV+Cocaine Mild Cognitive Impairment (MCMD)**	**Viral levels at Table 1**	↑ (IHC)	**NT**	↑ (IHC)	

b.

**Model**	**Treatments**	***HIV infection**	****Cathepsin B**	*****Apoptosis**	******Sig1R**	**Comments**
			In Vitro			
**MDM** 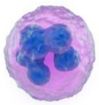	**HIV+**	↑	—	**NT**	**NT**	* p24 levels and ** CATB were tested from MDM supernatants by ELISA 11dpi
	**HIV+ Agonist**	—	—	**NT**	**NT**	
	**HIV+ Cocaine**	—	—	**NT**	**NT**	
	**HIV+Agonist /Cocaine**	—	—	**NT**	**NT**	

Supplementary Figure 11a: Raw Gel for CATB (above) and GAPDH expression (below) in HIVE Mice (1-2)

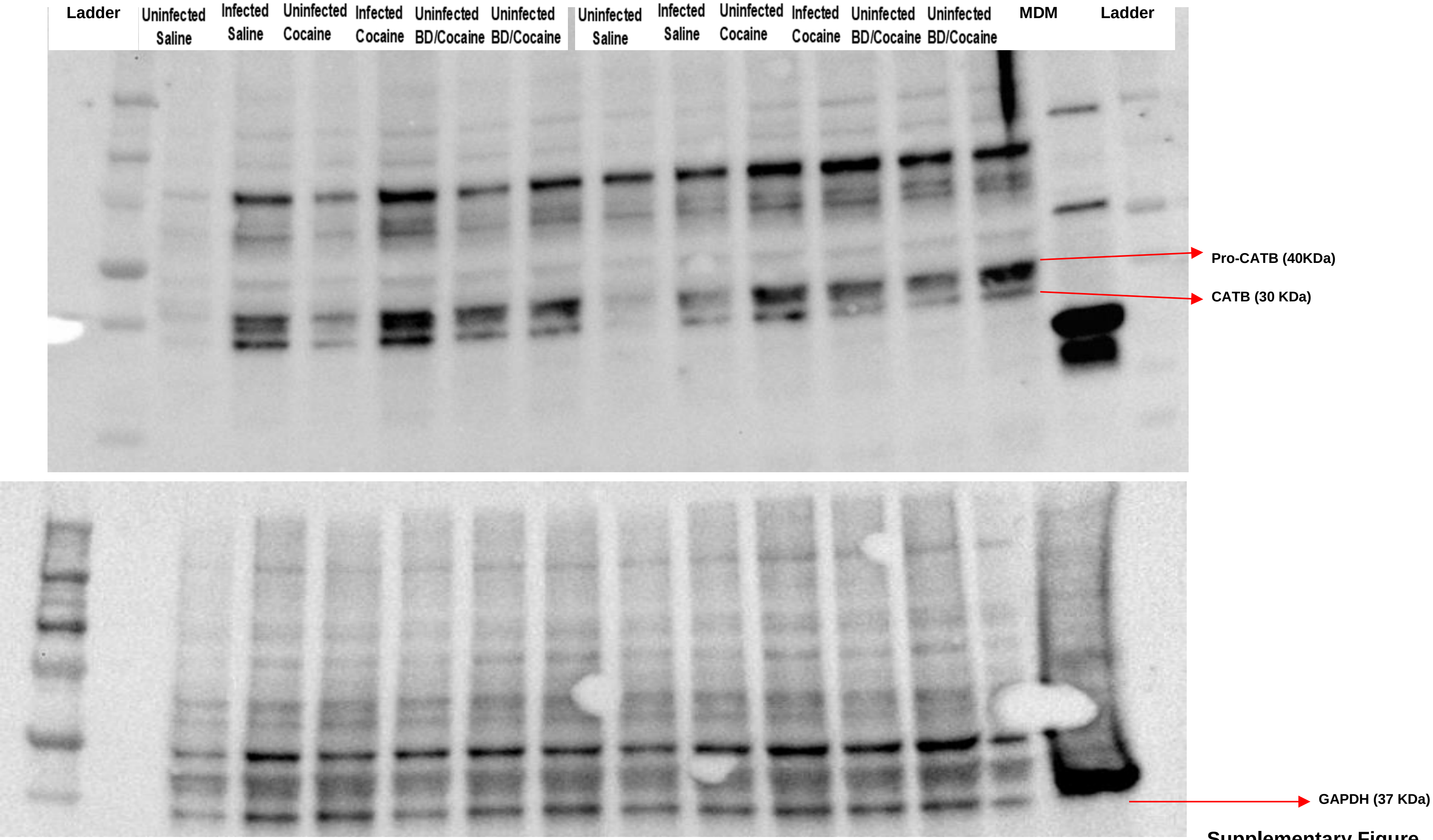

Supplementary Figure

11b: Raw Gel for CATB (above) and GAPDH (below) expression from HIVE Mice (3-4)

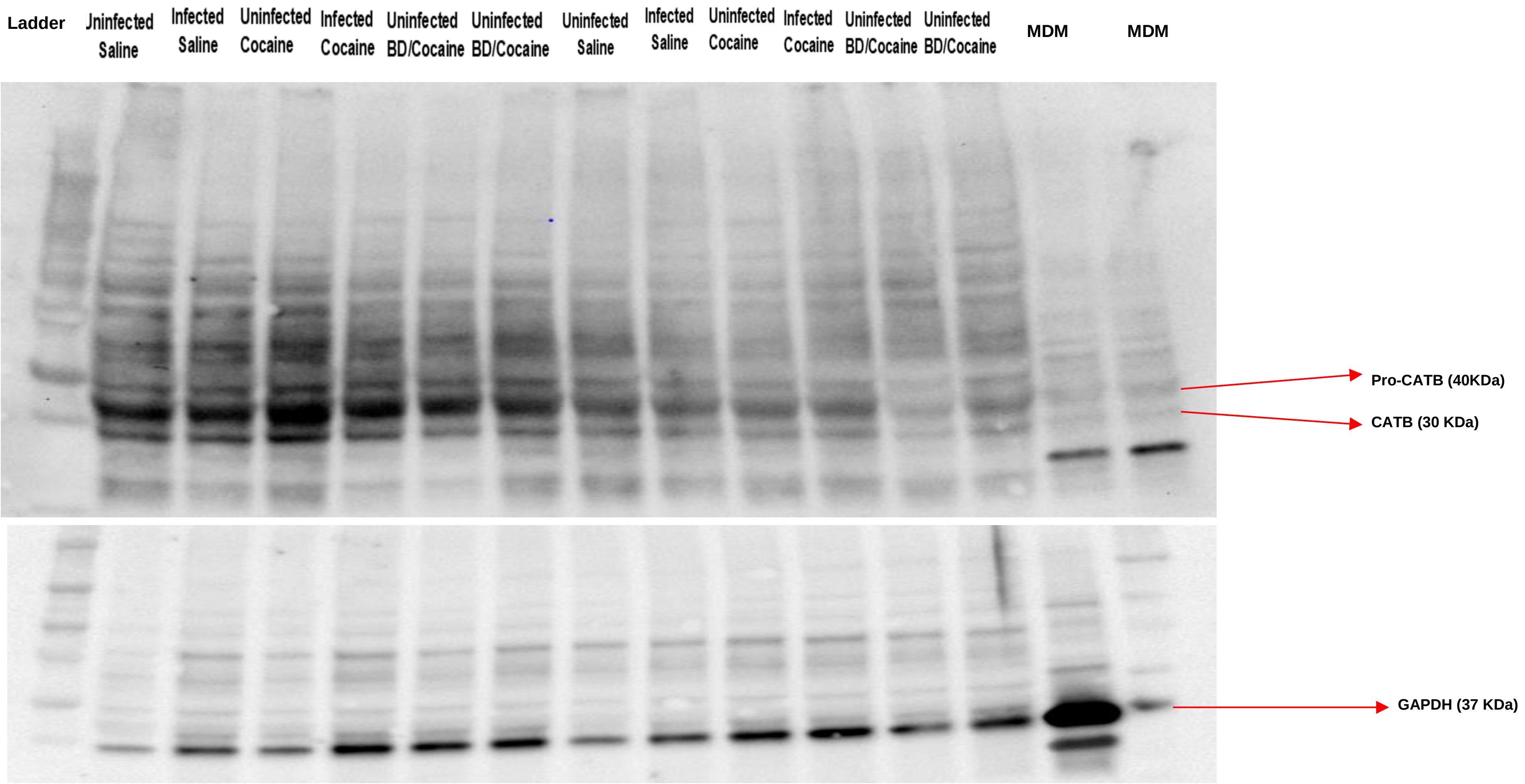

Supplementary Figure 11c: Raw Gel for Sig1R (above) and GAPDH (below) expression in HIVE Mice (1-2)

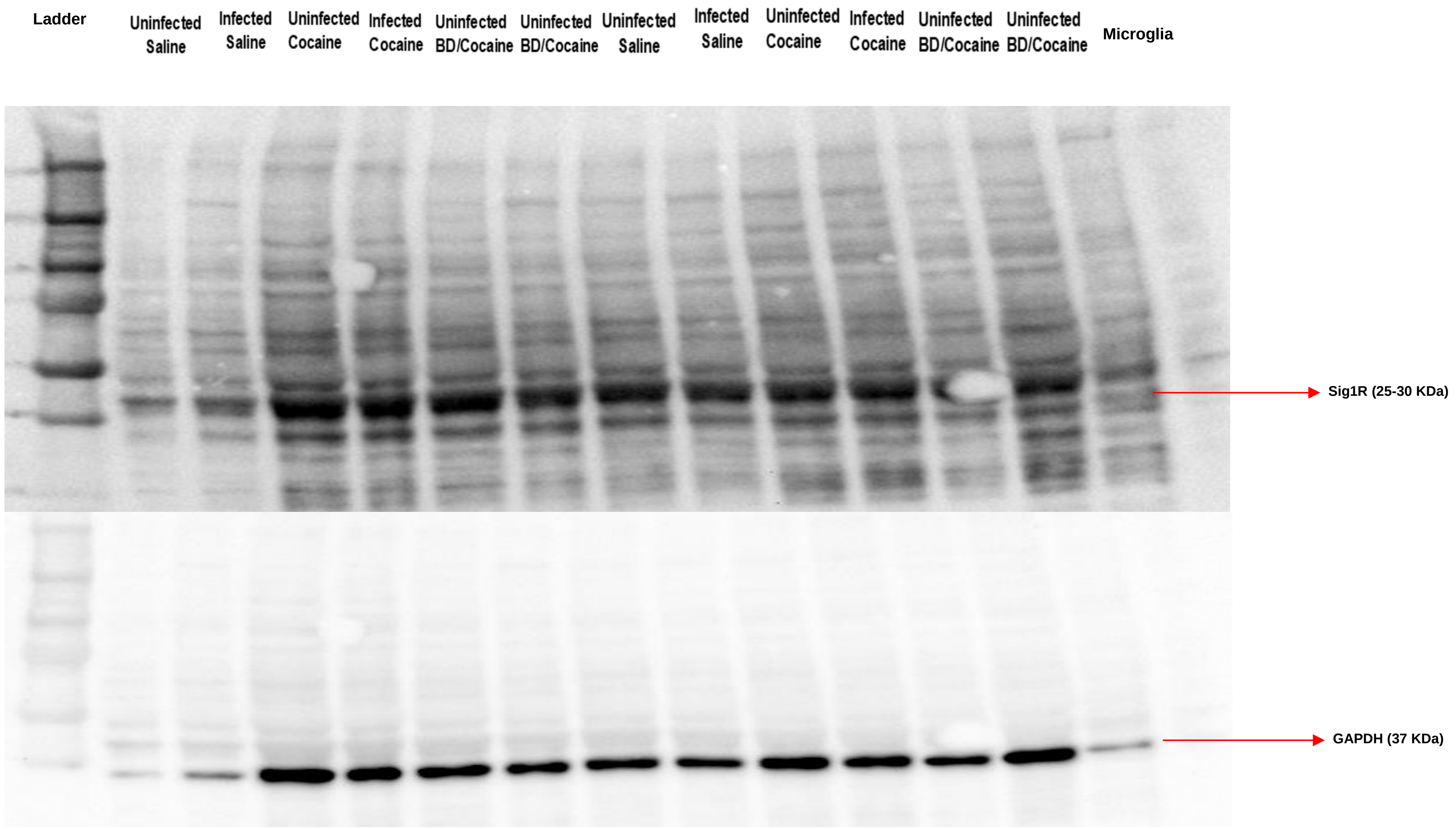

Supplementary Figure 11d: Raw Gel for Sig1R (above) and GAPDH (below) expression in HIVE Mice (3-4)

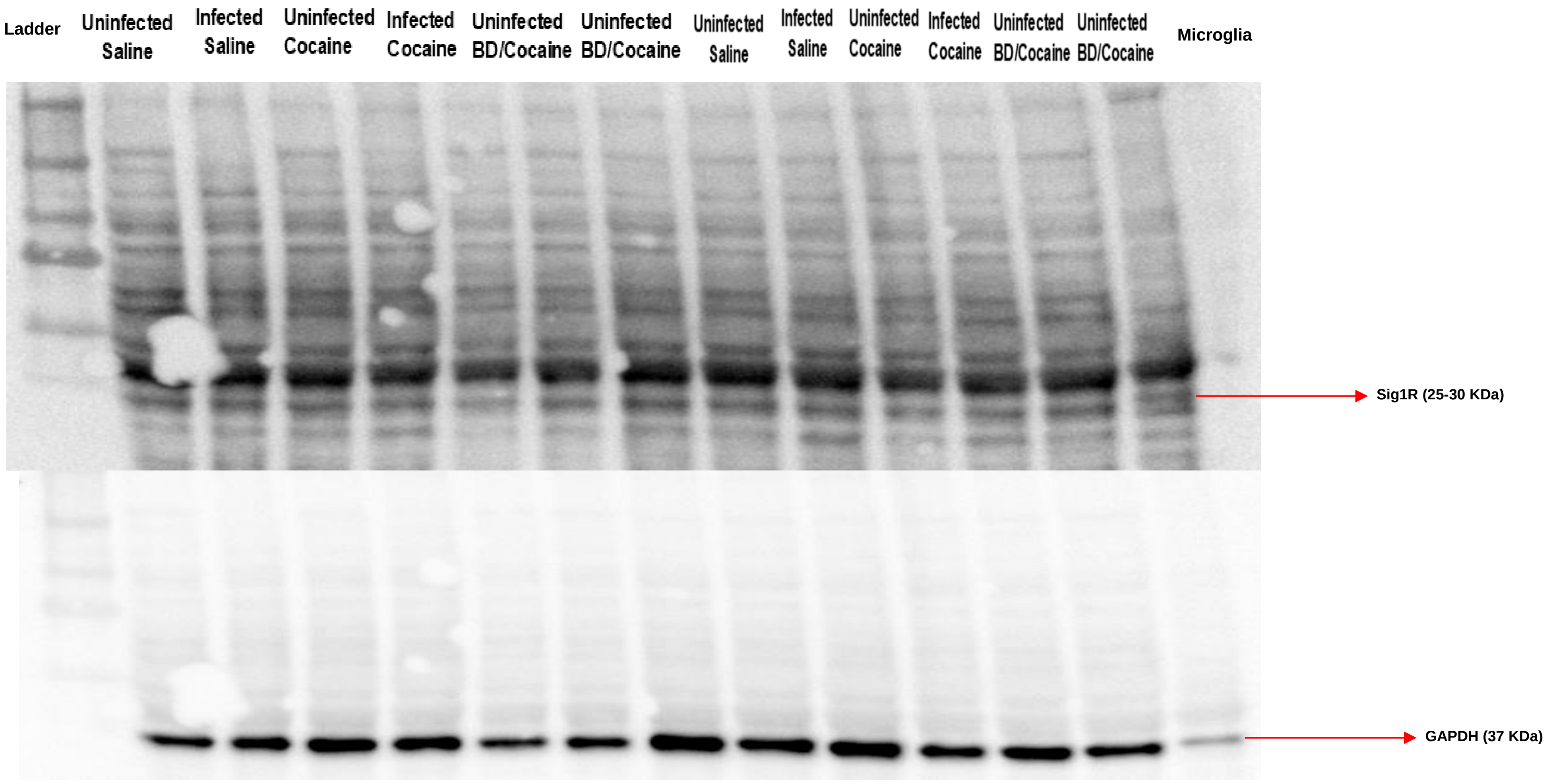

Supplementary Figure 11e: Raw Gel for MAP-2 (A/B & C/D) (above) and GAPDH (below) expression in HIVE Mice (n=3)

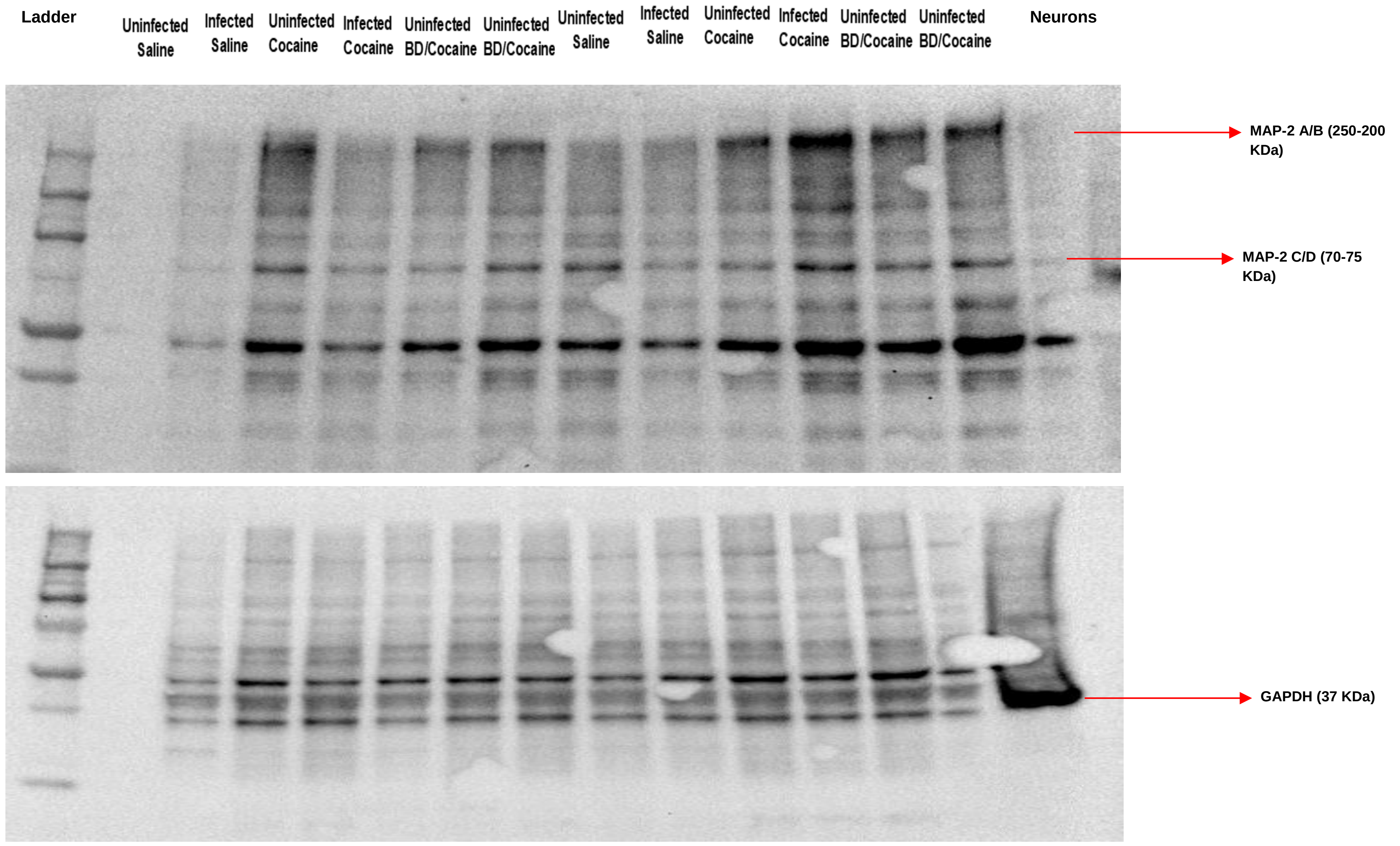

Supplementary Figure 11f: Raw Gel for MAP-2 (A/B & C/D) (above) and GAPDH (below) expression in HIVE mice (n=3)

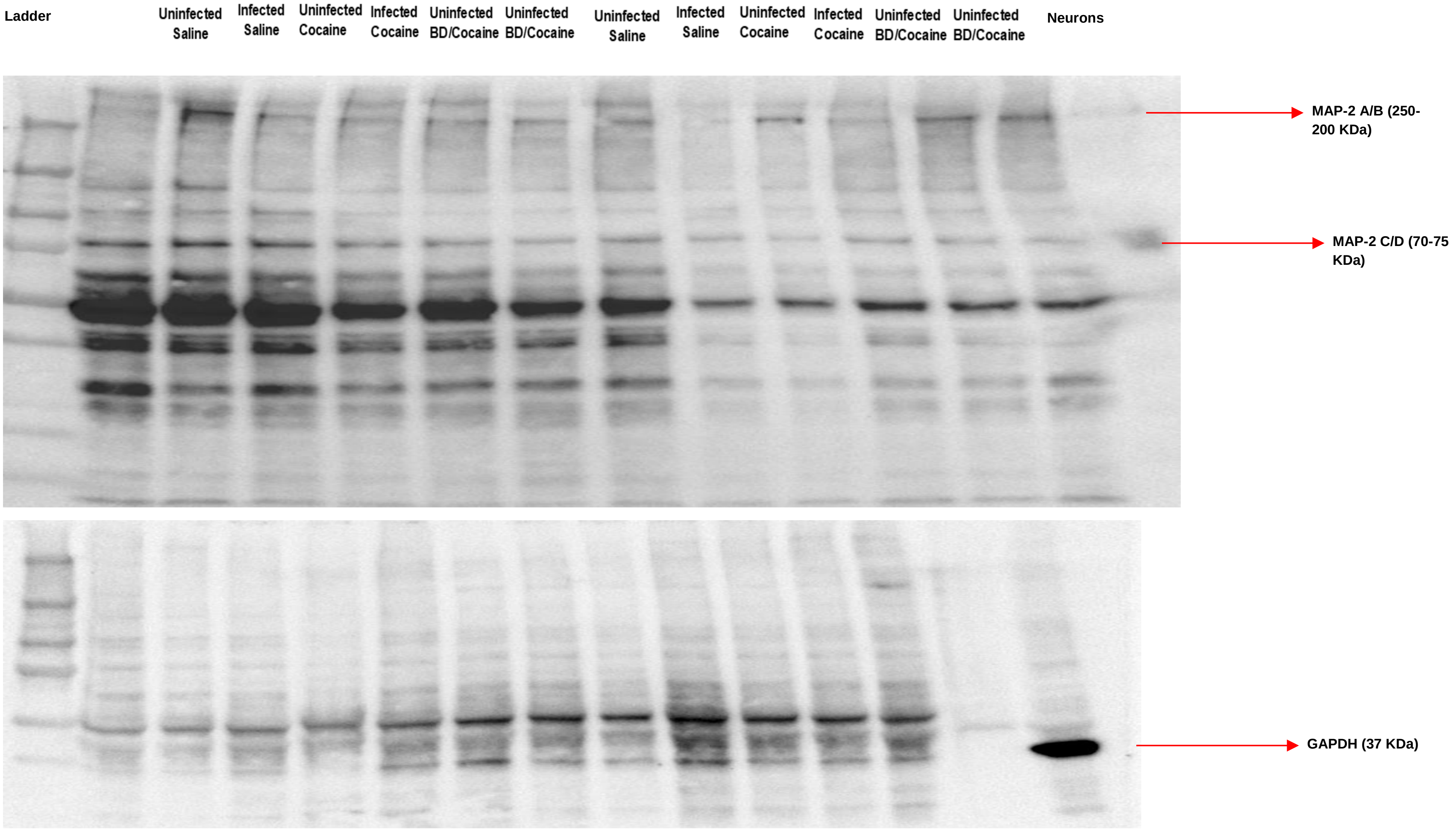

Supplement: Supplementary file 1 — (PDF 1520 kb) [file 11481_2018_9807_MOESM1_ESM.pdf]
